# Metal-Containing Borane and Hydrocarbon Sandwich Complexes: Exploring the 2D–3D Aromaticity Dilemma

**DOI:** 10.1021/acs.inorgchem.6c03817

**Published:** 2026-09-24

**Authors:** Zahra Noori, Clara Viñas, Miquel Solà, Francesc Teixidor, Jordi Poater

**Affiliations:** † Departament de Química Inorgànica i Orgànica & Institut de Química Teòrica i Computacional (IQTCUB), 16724 Universitat de Barcelona, Barcelona 08028, Spain; ‡ Institut de Ciència de Materials de Barcelona, Consejo Superior de Investigaciones Científicas, Bellaterra 08193, Spain; § Institut de Química Computacional i Catàlisi and Departament de Química, Universitat de Girona, Girona, Catalonia 17003, Spain; ∥ ICREA, Barcelona 08010, Spain

## Abstract

Recent reported planar metalloborane architectures raise the question of how aromaticity should be classified when conventional 2D π-aromatic ligands and boron-based frameworks capable of 3D multicenter delocalization are incorporated into structurally analogous metal complexes. Specifically, it remains unclear whether aromatic delocalization in these systems is predominantly localized within the ligands or extends through the metal to generate a three-dimensional (3D) framework. In this work, we employ DFT and a suite of complementary aromaticity indicators to analyze a series of sandwich complexes, including the recently reported CpOsB_6_H_11_ and [Os­(B_5_H_10_)_2_], in comparison with their hydrocarbon counterparts CpOsCp and ferrocene. For the systems investigated here, our results reveal markedly different degrees of metal integration into the aromatic framework. Coordination to the *nido*-borane frameworks produces enhanced global delocalization, greater metal–ligand multicenter delocalization, and more extensively coupled current-density pathways involving the metal. In contrast, the investigated cyclopentadienyl and related hydrocarbon complexes retain predominantly ligand-centered aromatic delocalization upon coordination, despite some degree of metal participation. On the basis of the combined magnetic, electron-delocalization, and orbital analyses, we propose a working classification ranging from predominantly ligand-centered 2D aromaticity to increasingly metal-integrated 3D aromaticity. These descriptions are not intended as rigid or universal categories, but rather identify the dominant aromatic-delocalization regime within the series examined.

## Graphical abstract

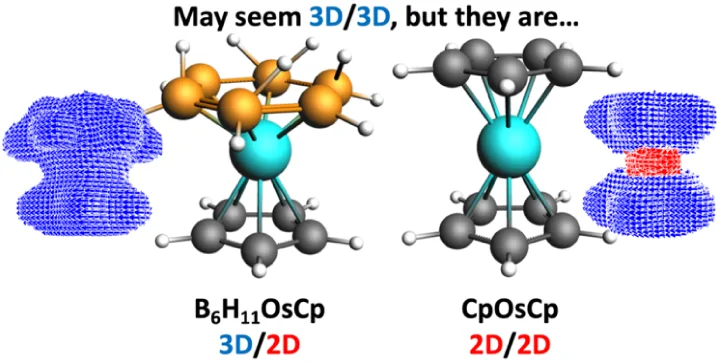

## Introduction

Wade’s rules provide a fundamental framework for understanding the structure and bonding of electron-deficient boron hydride clusters within cluster chemistry.^1,2^ In this context, *closo*, *nido*, and *arachno* boron clusters represent a systematic progression of polyhedral architectures governed by skeletal electron pair counts and vertex connectivity. *Closo* clusters, which have 2*n + 2* skeletal electrons (where *n* is the number of vertices in the polyhedron), adopt highly symmetrical, closed polyhedral geometries that serve as parent structures. In contrast, *nido* (2*n + 4* skeletal electrons) and *arachno* (2*n + 6* skeletal electrons) clusters arise from the removal of one and two vertices of a parent *closo*, respectively, resulting in increasingly open frameworks with reduced symmetry.^2–4^ This structural evolution leads to enhanced flexibility and the presence of accessible open faces, which are key features influencing their reactivity and coordination behavior. As a result, these cluster types play a central role in advancing the understanding of boron cluster bonding and continue to attract considerable attention in modern inorganic and organometallic chemistry.

Building on this structural paradigm, efforts to access nonclassical and less conventional boron frameworks—particularly planar motifs—have remained a significant challenge. In a very recent work, Ghosh, Jemmis, and coworkers have successfully achieved the synthesis of a planar hexaborane ring, a long-standing target in boron chemistry, referred to as the “boron analogue of benzene”.^5^ This challenge stems from boron’s inherent electron deficiency, which typically favors the formation of three-dimensional polyhedral structures rather than planar chains or rings. Consequently, earlier attempts to stabilize a planar B_6_ framework using monometallic templates were unsuccessful, although computationally Boldyrev et al. were able to planarize a B_6_ ring in Li_6_B_6_H_6_.^6^ It should be noted, however, that the occurrence of planar boron-containing rings in transition-metal complexes is not unprecedented. As early as 1977, Grimes and coworkers reported the synthesis of (η^5^-C_5_H_5_)­FeB_5_H_10_, a carbon-free analogue of ferrocene,^7,8^ which has recently been revisited through detailed quantum-chemical analysis by Oliva-Enrich and coworkers (Scheme 1).^9^ Notably, this compound has also been discussed in the recent study by Jemmis, Ghosh, and coworkers.^5^ Subsequently, Grimes and coworkers described a bimetallic complex featuring a perfectly planar five-membered C_2_B_3_ ring sandwiched between two cobalt centers (Scheme 1).^10^ Further striking examples were reported by Welch and coworkers, who identified 14-vertex bimetallic clusters of the type 1,14,2,10-RuM′C_2_B_10_ containing two fully planar six-membered CB_5_ layers capped by transition-metal fragments such as ruthenium (Scheme 1).^11^ Together, these studies demonstrate that transition-metal coordination can enforce planarity in electron-deficient boron frameworks, foreshadowing the recent isolation of the planar hexaborane ring.

**Scheme 1:**
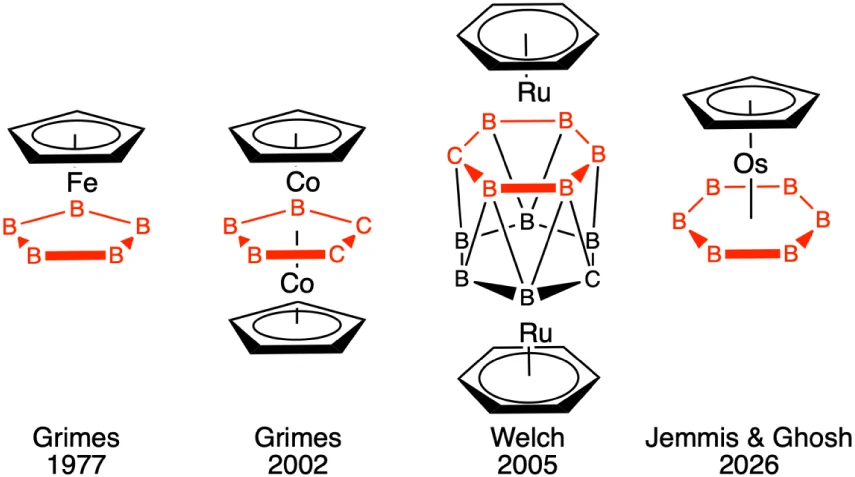
Previous Systems Studied by Grimes, Welch, and Jemmis and Ghosh^a^

a Planar boron rings in red.

In this context, Jemmis, Ghosh, and coworkers have reported the synthesis and characterization of bis-*nido*-[Cp*Os­(η^6^-B_6_H_11_)] (Cp* = [η^5^-C_5_Me_5_]^−^), representing the first example of a planar [B_6_H_11_]^−^ ring stabilized by a late transition metal fragment, [Cp*Os]^+^ (Figure 1). The compound exhibits notable fluxional behavior arising from rapid exchange of bridging hydrogens, while the planarity of the B_6_ ring is attributed to significant multicenter bonding interactions. As it is described, its aromatic character, supported by NICS analysis and frontier orbital similarities to bis­(benzene)­chromium, highlights an intriguing analogy between boron clusters and classical organic aromatic systems, despite the presence of σ–π mixing in the former.

**Figure 1:**
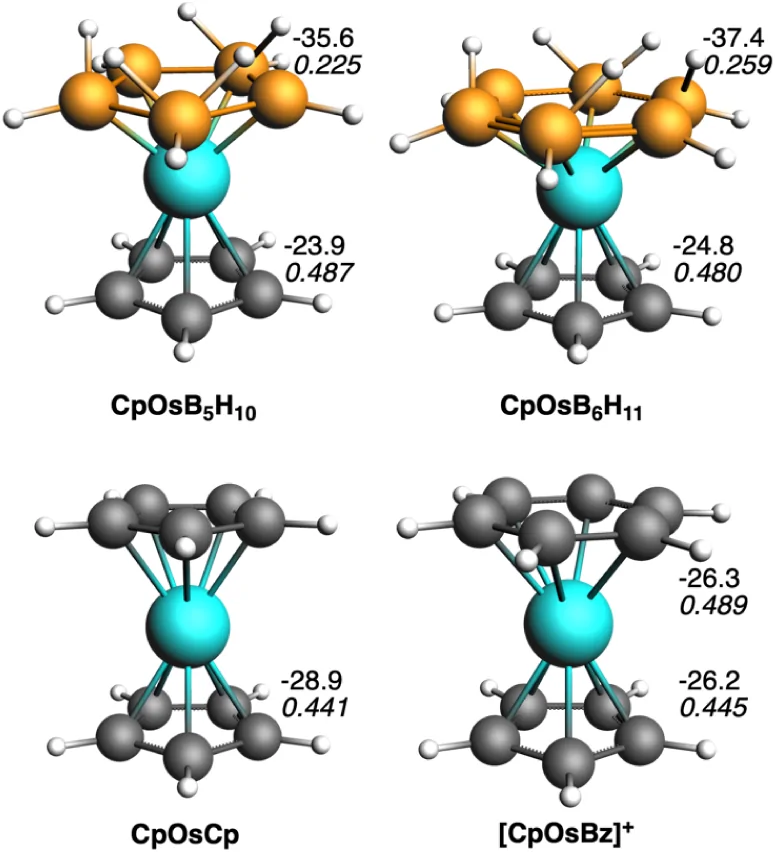
Wade’s equivalents to CpOsCp and [CpOsBz]^+^. NICS (computed at the center of rings, in ppm) and normalized MCI^1/N^ (*N* = number of members of the ring, in a.u., in italics) of boron and carbon rings. As reference systems: C_6_H_6_ (Bz, NICS = −7.4; MCI^1/N^ = 0.644), [C_5_H_5_]^−^ (Cp, NICS = −12.4; MCI^1/N^ = 0.586), [B_6_H_11_]^−^ (NICS = −1.0; MCI^1/N^ = 0.382), [B_5_H_10_]^−^ (NICS = −3.1; MCI^1/N^ = 0.394).

Despite the significance of the achievement of the synthesis of bis-*nido*-[Cp*Os­(η^6^-B_6_H_11_)], several fundamental questions remain open in order to fully rationalize the nature of this system and its comparison to conventional organic sandwich complexes. In particular, within the context of Wade’s rules, it is not yet clear whether CpOsCp can be considered electronically equivalent to CpOsB_6_H_11_ (experimentally studied Cp* and computational Cp gives equivalent results). While we are accustomed to the fact that in metallocene sandwich or half-sandwich complexes, the planarity of the arene is preserved due to its aromaticity, this is in stark contrast to boranes, where their planarity is hindered by a tendency to cluster. Therefore, the true extent and origin of the aromaticity of the planar hexaborane ring requires further clarification. The role of the osmium center is central to the discussion. Beyond serving as a stabilizing fragment, it is essential to elucidate how its interaction with the B_6_ framework differs from that with classical ligands such as cyclopentadienyl anion and whether distinct bonding regimes are operative. This analysis aims to address also whether metallocenes, such as ferrocene, Fe­(C_5_H_5_)_2_, are electronically equivalent to compounds like FESAN, [Fe­(C_2_B_9_H_11_)_2_]^−^, or alternatively whether FESAN is a sandwich complex or a cluster. Beyond the assignment of aromatic character itself, these systems raise a more fundamental question: do the formal electronic and structural analogies established by Wade’s rules and the Electronically Confined Space Analogy (ECSA) also imply analogous modes of aromatic delocalization? In particular, it remains unclear whether a transition metal plays an equivalent electronic role when interacting with a conventional hydrocarbon π ligand and with a planar electron-deficient borane framework. Addressing this question requires going beyond individual magnetic descriptors and determining whether the metal remains external to a predominantly ligand-centered aromatic circuit or instead becomes electronically integrated into a multicenter delocalized framework. Here, by combining current-density and electron-delocalization analyses with molecular orbital and energy decomposition analyses, we show for the investigated systems that these formally analogous complexes display fundamentally different modes of metal–ligand electronic integration. This provides a chemical basis for distinguishing ligand-centered 2D aromaticity from metal-integrated 3D aromaticity and establishes that structural and electron-counting analogies between hydrocarbon and borane complexes do not necessarily translate into equivalent aromatic behavior. More generally, our analysis tests whether formal electronic equivalence between hydrocarbon and borane fragments also entails equivalent metal–ligand bonding and aromatic dimensionality, thereby connecting aromaticity to the underlying nature of metal–ligand electronic integration.

## Results and Discussion

Back to Wade’s rules, these electron-counting principles provide a useful framework to establish analogies between classical organometallic fragments and boron clusters within cluster chemistry. According to this approach, the fragment CpOsCp can be related to a boron analogue in which the osmium fragment, [CpOs]^+^, formally replaces a cluster vertex. Under this assumption, the species electronically equivalent to CpOsCp would be CpOsB_5_H_10_, where the remaining Cp ligand is replaced by a [B_5_H_10_]^−^ fragment, which together with the metal vertex gives rise to a *nido* boron cluster (Figure 1). This system was first synthesized by Grimes and coworkers.^7,8^

A similar reasoning can be extended to [CpOsBz]^+^. If [CpOs]^+^ is again considered as a single vertex contributor, the benzene ligand (Bz) can be mapped onto a [B_6_H_11_]^−^ unit, leading to the formulation CpOsB_6_H_11_, which also corresponds to a *nido* boron cluster. In both cases, the resulting structures can be interpreted as *nido* clusters formed by the combination of a planar boron fragment with an osmium unit that completes the corresponding pentagonal or hexagonal pyramidal framework, yielding CpOsB_5_H_10_ and CpOsB_6_H_11_, respectively (Figure 1).

The above equivalences derived from Wade’s rules can also be considered within the framework of the Electronically Confined Space Analogy (ECSA), which relates molecular fragments that occupy comparable confined regions of space and possess not only appropriate electron counts but also compatible frontier-orbital characteristics that allow analogous interactions with their surroundings.^12–14^ In the present context, ECSA is used as a conceptual framework for rationalizing the formal relationship between the hydrocarbon and borane fragments rather than as evidence for equivalent bonding or aromatic behavior.

Importantly, these underlying planar boron fragments are electronically saturated, lacking low-lying vacant orbitals capable of supporting conventional delocalized π bonding. On this basis, one might anticipate that neither [B_5_H_10_]^−^ nor [B_6_H_11_]^−^ possesses intrinsic aromatic character, raising important questions regarding the origin and validity of the aromaticity assigned to the corresponding metal-stabilized systems. However, at this point, we must refer to a very recent study by Merino and Tiznado showing that the planar isomer of [B_5_H_10_]^−^ lies 78.2 kcal mol^–1^ above its nonplanar global minimum,^15^ demonstrating that the planar ring requires metal coordination to become thermodynamically accessible. This situation reminds that of cyclobutadiene metal complexes.^16^ Despite the intrinsic instability of planar [B_5_H_10_]^−^, AdNDP analysis revealed substantial electron delocalization, identifying three 15-center–2-electron π-type orbitals. Moreover, the computed diatropic magnetically induced current density provides evidence of global electron delocalization, supporting the assignment of aromatic character to this planar boron ring.

First, although computed NICS values suggest a pronounced aromatic character for both [B_5_H_10_]^−^ and [B_6_H_11_]^−^ rings within the clusters (−35.6 and −37.4 ppm, respectively, Figure 1), this effect can be primarily attributed to the presence of the metal center. Indeed, coupling between the ring currents of osmium and those of the boron frameworks leads to artificially enhanced diatropic responses. In contrast, the isolated planar boron fragments exhibit essentially nonaromatic behavior, as reflected by much less negative NICS values (−3.1 and −1.0 ppm, respectively; Figure 1). Consistently, current-density maps reveal the absence of a continuous diatropic ring current in the free species, with clear interruptions at B–B edges lacking bridging hydrogen atoms (Figure S1). DIAL isosurfaces further corroborate the crucial role of these bridging hydrogens in sustaining local current pathways. And for completeness, the computed electronic-based normalized multicenter values (MCI^1/N^) also show the low aromaticity of these boron rings (0.259 and 0.225 au for CpOsB_6_H_11_ and CpOsB_5_H_10_, respectively). These values are even smaller than in the isolated boron fragments (0.394 and 0.382 au, respectively), which are already far from those of either benzene (0.644 au) or Cp^–^ (0.586 au). Importantly, these MCI^1/N^ values agree with the certain degree of electronic delocalization shown by the planar [B_5_H_10_]^−^ by means of AdNDP.^15^

Upon coordination to osmium, however, the induced current density generates a strong and continuous diatropic ring current over the boron framework, with significant bond current strengths of 77 and 59 for the B_6_ and B_5_ rings, respectively (Figure 2); as a reference, the value of the bond current strength in C_6_H_6_ is 100. For comparison, bond current strengths for [B_6_H_11_]^−^ ring alone amount to 28–32, whereas those for [B_5_H_10_]^−^ ring alone to only 16 (the five bonds are equivalent, and for B–H bonds the bond current strength is 15). It should be emphasized that these relatively low bond current strengths do not contradict the global electron delocalization identified for planar [B_5_H_10_]^−^ by AdNDP and induced-current analyses.^15^ The bond current strength provides a local, bond-resolved measure of the magnitude of the current crossing a particular B–B region, whereas global aromatic circulation refers to the continuity and topology of the induced current throughout the entire ring. Thus, a continuous delocalized current pathway may coexist with relatively weak currents across individual B–B bonds. In this sense, the value obtained for planar [B_5_H_10_]^−^ indicates that global electron delocalization is already present in the free ligand, although substantially weaker than in benzene and markedly enhanced upon coordination to the metal.

**Figure 2:**
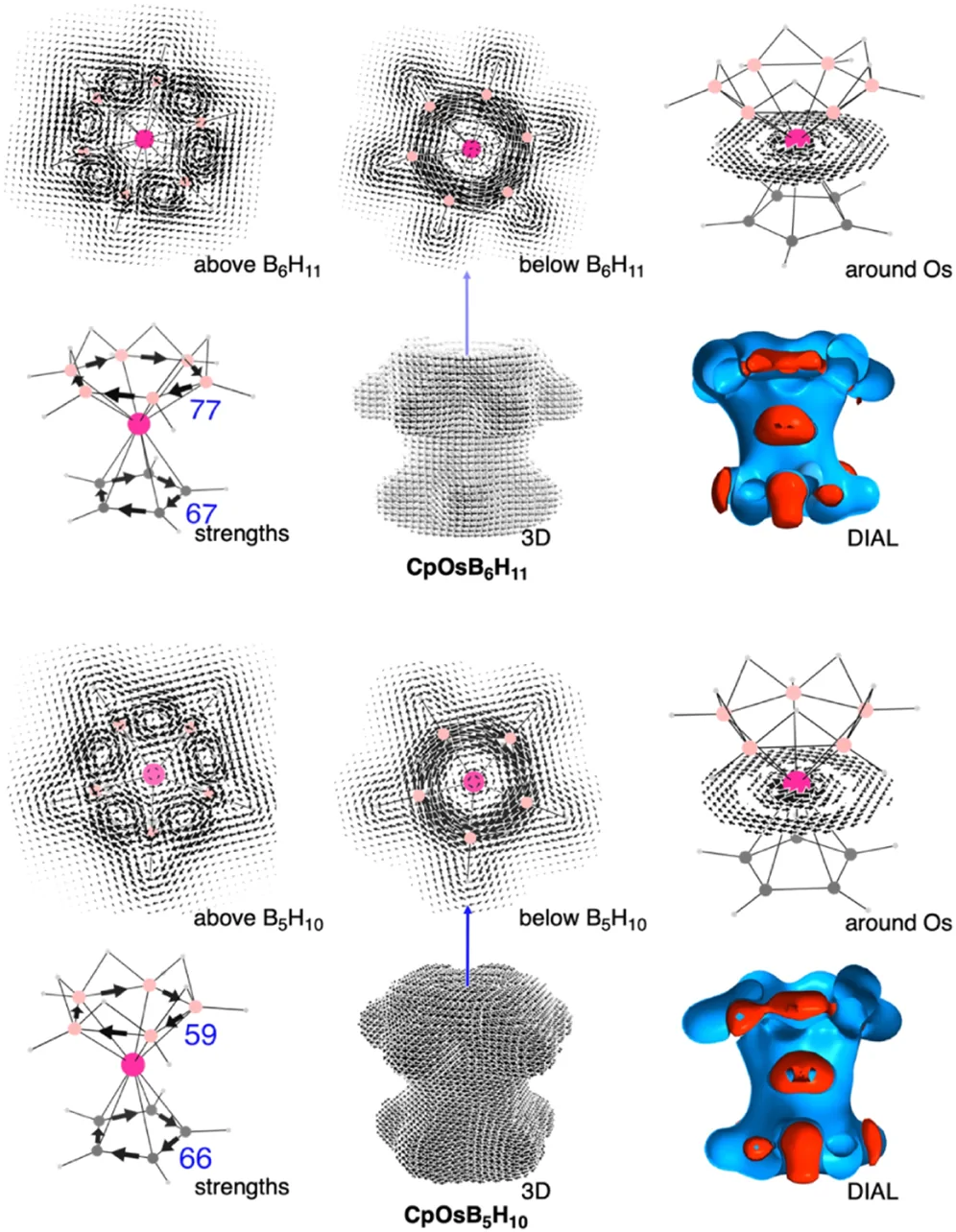
Current density maps of CpOsB_6_H_11_ and CpOsB_5_H_10_. Clockwise/anticlockwise circulation corresponds to diatropic/paratropic ring currents. For the ligands, the ring currents are generated at 1 a.u. above and below the ring plane. In the DIAL representation, diatropic regions are shown in blue and paratropic in red. Bond current strengths are given as percentages relative to a reference value of 12 nA T^–1^.

Also notably, this current is not confined to the boron rings but extends along the *z*-axis through the osmium center and into the cyclopentadienyl ligands (Cp), thus giving rise to a global delocalized pathway across the complex. This metal-induced diatropic circulation is particularly evident in the region between the boron ring and the osmium center, i.e., below the ring plane, where a pronounced current density is observed, together with a strong diatropic component in the plane of the metal. In contrast, in the region above the boron ring, outside the cluster framework, the current density becomes localized rather than delocalized (Figures S1 and S2), similar to those found in free [B_6_H_11_]^−^ and [B_5_H_10_]^−^ ligands. These localized currents can be directly associated with B–H–B interactions involving the bridging hydrogen atoms, highlighting their role in modulating local electron delocalization while not contributing to a continuous global ring current.

In contrast, such a metal-induced enhancement is not observed for the cyclopentadienyl ligand. On the contrary, its bond current strength slightly decreases upon coordination, from 70 in the free [C_5_H_5_]^−^ ligand to 67 and 66 in CpOsB_6_H_11_ and CpOsB_5_H_10_, respectively. This trend is at odds with the corresponding NICS values, which instead suggest an apparent increase in aromaticity upon coordination (from −12.4 in the free ligand to −24.8 and −23.9 in CpOsB_6_H_11_ and CpOsB_5_H_10_, respectively). Further insight is again provided by the normalized multicenter index, which indicates a reduction in intrinsic aromaticity, decreasing from 0.586 au in the free Cp^–^ ligand to 0.480 and 0.487 au in CpOsB_6_H_11_ and CpOsB_5_H_10_, respectively (Figure 1). Taken together, these results highlight that NICS values, when applied to metal-containing systems, may predominantly reflect metal-induced magnetic effects rather than genuine ring aromaticity, and should therefore be interpreted with caution.^17^

The above discussion of the aromaticity of CpOsB_6_H_11_ has been carried out considering cyclopentadienyl anion as ligand; however, the experimental system employs pentamethylcyclopentadienyl anion (Cp*). The introduction of the electron-donating methyl substituents leads to a slight decrease in aromaticity, not only in the substituted C_5_ ring but also in the B_6_ framework (Figure 3). This effect can be attributed to the increased electron density provided by the methyl groups, which perturbs the metal–ligand interactions and, consequently, the extent of delocalization across the system.

**Figure 3:**
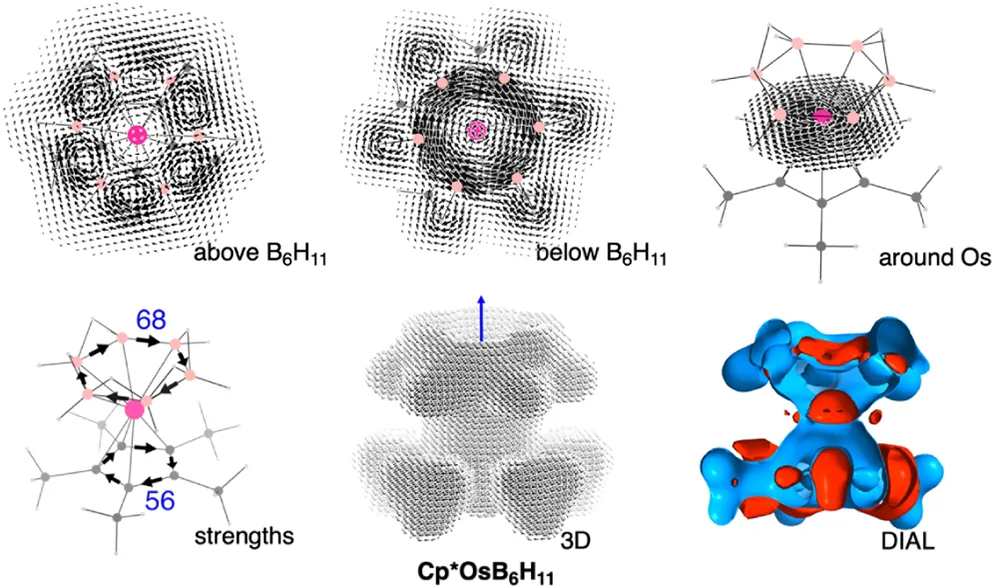
Current density maps of Cp*OsB_6_H_11_. Clockwise/anticlockwise circulation corresponds to diatropic/paratropic ring currents.For the ligands, the ring currents are generated at 1 a.u. above and below the ring plane. In the DIAL representation, diatropic regions are shown in blue and paratropic in red. Bond current strengths are given as percentages relative to a reference value of 12 nA T^–1^.

Once the aromaticity of CpOsB_6_H_11_ and CpOsB_5_H_10_ has been analyzed, attention can be turned to their hydrocarbon counterparts derived from Wade’s rules, namely [CpOsBz]^+^ and CpOsCp (Figure 4). Notably, the extended diatropic ring current previously observed—from the boron framework through the osmium center and into the cyclopentadienyl ligand—is absent in these carbon-based analogues. Instead, a key distinguishing feature is the presence of a paratropic ring current around the osmium center, clearly revealed by the three-dimensional isosurfaces of the current density maps. Consistent with this observation, the influence of the metal on the aromaticity of the hydrocarbon ligands is comparatively minor. The bond current strengths for both cyclopentadienyl and benzene remain largely unaffected upon coordination. For instance, the bond current strength of benzene decreases only slightly, from 100 in the free molecule to 89 in [CpOsBz]^+^. This behavior contrasts sharply with that found in the boron systems, for which the bond current strength increases upon coordination to osmium.

**Figure 4:**
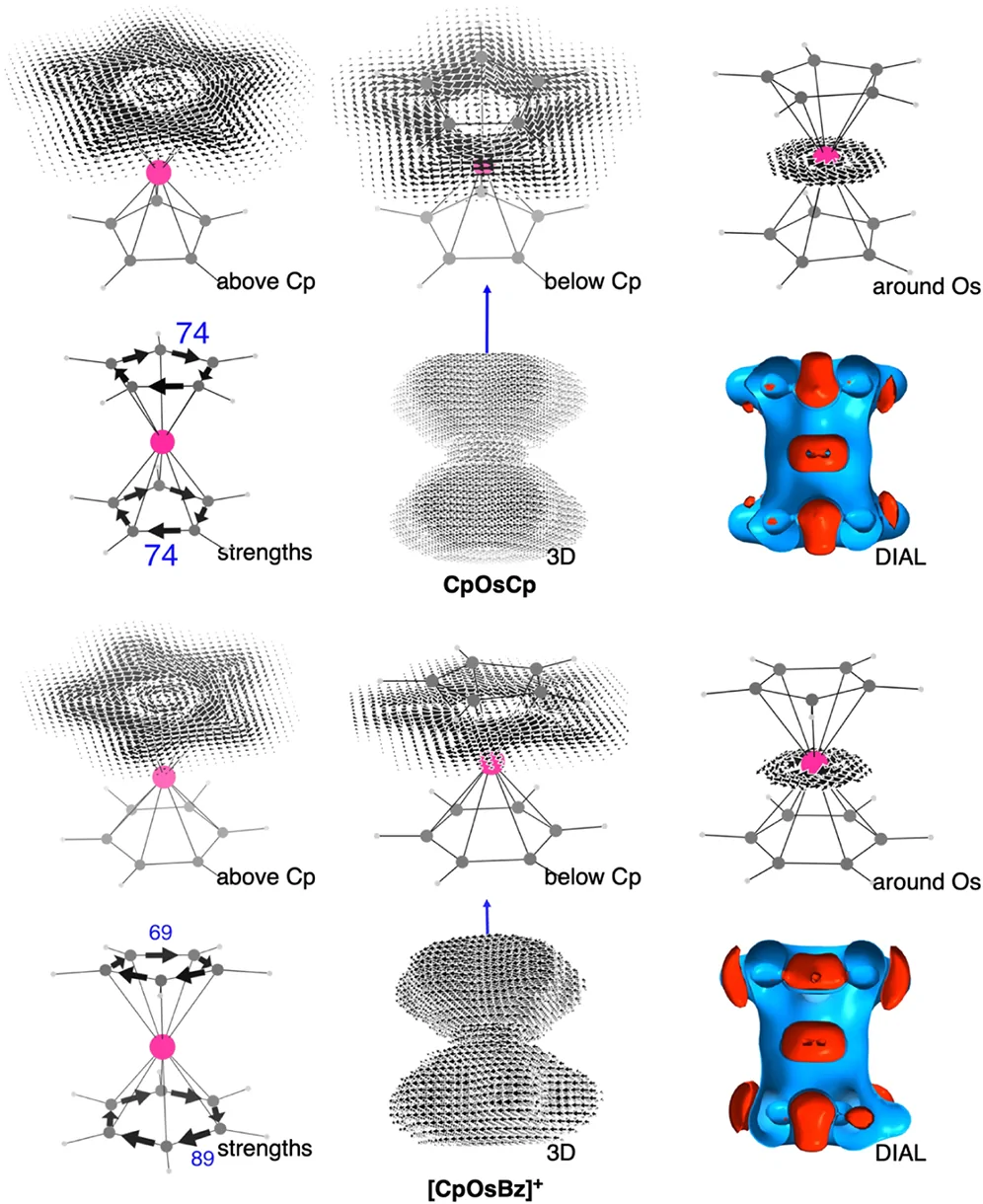
Current density maps of CpOsCp and [CpOsBz]^+^. Clockwise/anticlockwise circulation corresponds to diatropic/paratropic ring currents. For the ligands, the ring currents are generated at 1 a.u. above and below the ring plane. In the DIAL representation, diatropic regions are shown in blue and paratropic in red. Bond current strengths are given as percentages relative to a reference value of 12 nA T^–1^.

A plausible explanation for these differences lies in the distinct role played by osmium in each case. In the boron clusters, the metal is effectively incorporated into the cluster framework, in most of the cases leading to *nido* 3D-aromatic system.^18^ By contrast, in the hydrocarbon analogues, osmium does not become an integral part of the π-delocalized framework and the aromaticity is concentrated in the ligands. This interpretation is supported by molecular orbital analysis: in CpOsB_6_H_11_, there is substantial mixing between the osmium *d* orbitals and the boron framework, leading to multicenter Os–B bonding interactions (Figure S2). In contrast, such mixing is far less pronounced in [CpOsBz]^+^, where the interaction between the metal and the π systems of cyclopentadienyl or benzene remains more localized and less cooperative (Figure S2).

It is worth mentioning that the decrease in the ring-based MCI upon coordination referred above, despite the pronounced enhancement of the diatropic current, indicates that coordination should not be interpreted as a uniform increase in the aromaticity of the borane ring itself. Rather, ring-localized electron delocalization is partially redistributed into a broader metal–borane framework, consistent with the extensive Os–B orbital mixing. Thus, the magnetic enhancement and decrease in ring-localized MCI are complementary rather than necessarily contradictory manifestations of the electronic reorganization induced by metal coordination.

This latter interpretation is in line with our previous findings, where we demonstrated that metallabis­(dicarbollides) exhibit global aromaticity, whereas sandwich metallocenes display aromaticity localized on the ligands.^18^ Accordingly, in CpOsB_6_H_11_, the interaction between osmium and the boron ring closely resembles that found in metallabis­(dicarbollides), giving rise to a three-dimensional aromatic system.^19^ In this case, osmium plays a key structural and electronic role, interacting with an initially nonaromatic two-dimensional boron fragment and promoting its transformation into an aromatic *nido* cluster. In contrast, the interaction between osmium and the Cp ligand in the same complex is better described in terms of metallocene-like bonding, where aromaticity remains localized on the hydrocarbon ring and the metal participate significantly less in the delocalized π-system.

Thus, according to the above definition, CpOsB_6_H_11_ can therefore be described as a mixed 2D/3D aromatic system. On the Cp side, aromatic delocalization remains predominantly confined to the hydrocarbon ring, which largely preserves the characteristics of the free Cp^–^ ligand and is therefore classified as 2D aromatic. On the borane side, however, extensive Os–B orbital mixing and current pathways involving both the metal and the boron framework indicate that Os becomes an integral component of the delocalized framework, consistent with a 3D aromatic OsB_6_H_11_ unit. The term “mixed 2D/3D” thus describes the coexistence of these two distinct delocalization regimes within the same molecular structure.^20–25^

Further insight into the distinct interactions in CpOsB_6_H_11_ and CpOsB_5_H_10_ relative to their hydrocarbon counterparts is provided by quantitative Kohn–Sham molecular orbital and energy decomposition analyses. In the case of CpOsB_5_H_10_, the interaction between osmium and the boron ring is significantly stronger than that with the cyclopentadienyl ligand (Δ*E*_int_ = −335.0 vs −220.9 kcal mol^–1^; Table S1). This observation supports the view that osmium effectively becomes part of the *nido* cluster in interaction with the B_5_ framework, whereas its bonding with Cp resembles that found in classical systems such as ferrocene. Energy decomposition analysis further reveals that, although the Os–boron interaction is associated with increased Pauli repulsion, this repulsion is more than compensated by stronger electrostatic and orbital interaction terms. Moreover, the enhanced contribution of the Δ*E*_oi_ term indicates a higher degree of covalent character, in line with the pronounced mixing between osmium *d* orbitals and the boron framework discussed above. Noticeably, this stronger Os–borane interaction and pronounced orbital mixing support a greater degree of electronic integration of the metal into the borane framework. These bonding features are consistent with, but do not by themselves establish, the assignment of 3D aromaticity, which relies on their combined interpretation with the magnetic and electron-delocalization analyses.

Importantly, the 2D/2D, mixed 2D/3D, and 3D/3D terminology used here should not be interpreted as defining rigid or mutually exclusive categories. Rather, these descriptions constitute a working classification of the dominant topology of aromatic delocalization within the systems investigated. The degree of metal participation can vary continuously as a function of metal–ligand orbital matching and electronic structure, giving rise to a continuum ranging from predominantly ligand-centered 2D aromaticity to extensively metal-integrated 3D multicenter delocalization. The labels employed here identify the dominant regime along this continuum rather than implying sharp boundaries between distinct forms of aromaticity. We use predominantly 2D aromaticity to describe systems in which aromatic delocalization remains largely ligand-centered, even though some metal participation in the induced current may occur, as illustrated by ferrocene. In contrast, the characteristics of 3D aromaticity arise when the metal becomes an integral component of a multicenter delocalized framework, as supported by both electronic-delocalization and current-density analyses. Accordingly, the assignments made here rely on the combined analysis of current-density topology, multicenter electron delocalization, and metal–ligand orbital mixing and are restricted to the systems examined in the present study. For such purpose, and for ease of comparison, the principal NICS, normalized MCI, and bond current strength values for the above investigated systems are collected in Table 1.

**Table 1:** Summary of NICS (in ppm ), MCI^1/N^ (in a.u.) and Bond Current Strengths (BCS, in Percentage Relative to a Reference Value of 12 nA T^–1^) of the Main Systems under Analysis

System	Ring	NICS	MCI^1/N^	BCS
CpOsB_6_H_11_	B_6_	–37.4	0.259	77
	C_5_	–24.8	0.480	67
CpOsB_5_H_10_	B_5_	–35.6	0.225	59
	C_5_	–23.9	0.487	66
[CpOsBz]^+^	C_6_	–26.3	0.489	89
	C_5_	–26.2	0.445	69
CpOsCp	C_5_	–28.9	0.441	74
CpRuCp	C_5_	–29.9	0.447	75
CpFeCp	C_5_	–37.9	0.446	81

Notably, the interaction between osmium and the Cp^–^ ligand in CpOsB_5_H_10_ closely resembles that found in its hydrocarbon analogue CpOsCp, where the Os–cyclopentadienyl interaction amounts to −242.5 kcal mol^–1^ (Table S1). And this interaction is much smaller than that between [CpOs]^+^ + [B_5_H_10_]^−^ fragments (−335.0 kcal mol^–1^, Table S1). This similarity is further supported by fragment molecular orbital (FMO) analysis (Figure 5), which in both cases reveals a donor–acceptor interaction between the *d* orbitals of osmium and the *p*_*z*_ orbitals of the Cp ligand. However, the extent of charge transfer from Cp^–^ to the metal is more pronounced in CpOsCp, consistent with its larger orbital interaction term in the energy decomposition analysis (−204.2 vs −163.6 kcal mol^–1^ for CpOsCp and CpOsB_5_H_10_, respectively). This trend is also reflected in the populations of the metal-centered LUMO, LUMO+1, and LUMO+2 orbitals, which are higher in CpOsCp (1.50 e) than in CpOsB_5_H_10_ (1.25 e), indicating a stronger donor–acceptor interaction in the former system.

**Figure 5:**
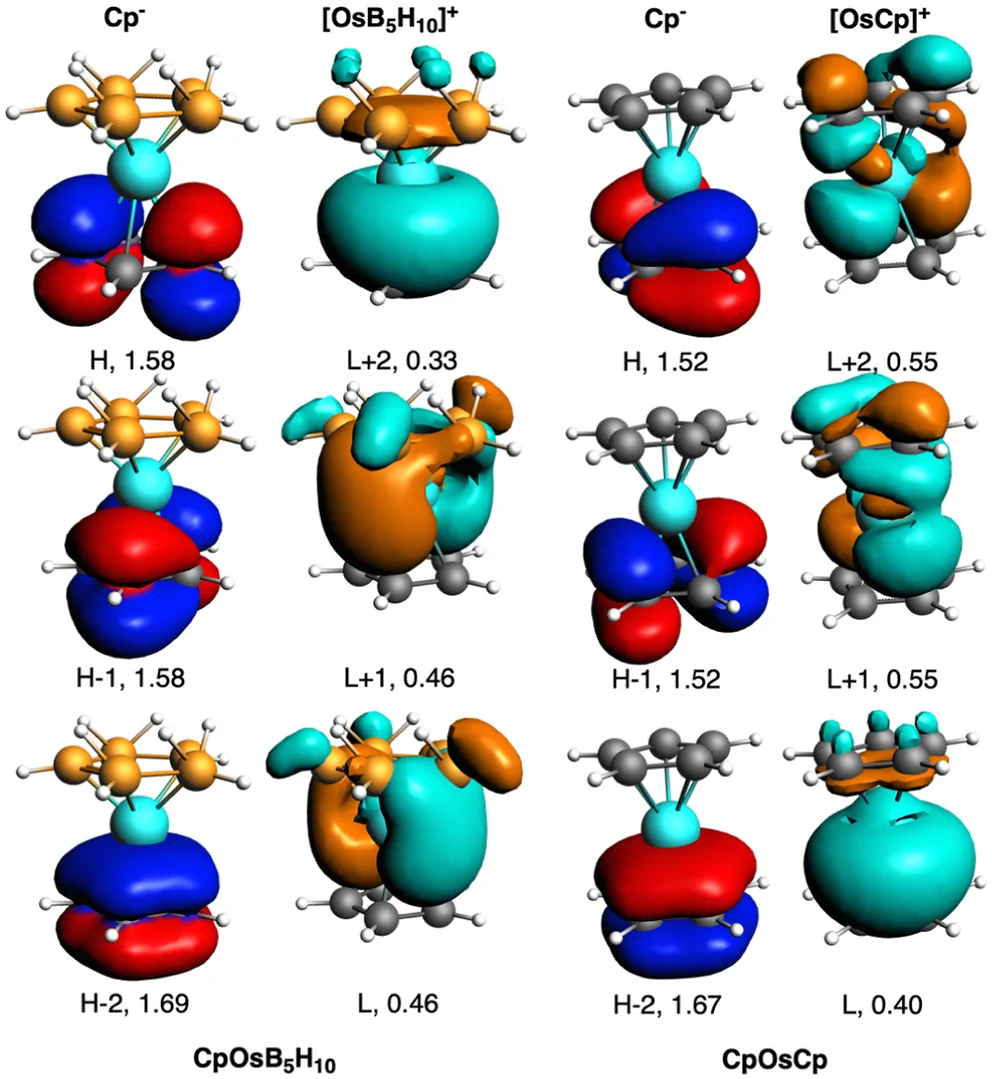
Molecular orbitals involved in the main donor–acceptor interaction of CpOsB_5_H_10_ (left) and CpOsCp (right) considering the breaking between the osmium-ligand and the cyclopentadienyl anion, with the corresponding Gross Mulliken populations included (in electrons). Left orbitals transfer charge to right orbitals in each case, while the rest of the molecular orbitals are not depicted for simplicity. H and L refer to HOMO and LUMO, respectively.

In contrast, such a well-defined unidirectional charge transfer from cyclopentadienyl anion to the metal is not observed for the Os–boron interaction in CpOsB_5_H_10_ (Figure S3). Instead, a pronounced mixing between the fragment molecular orbitals of both fragments—namely the boron ring and the osmium center—is evident. In this case, donor–acceptor interactions occur in both directions (donation and backdonation), leading to partially occupied LUMO orbitals on both fragments. The FMOs of the [CpOs]^+^ fragment are predominantly metal-centered, arising mainly from osmium *d* orbitals, with only a minor contribution from the Cp ligand. In contrast, the FMOs of the boron ring are composed of boron *p* and hydrogen *s* orbitals, giving rise to orbital patterns that bear some resemblance to the π-system of benzene, despite originating from three-center–two-electron bonding (Figure S3).

The above comparison between CpOsB_5_H_10_ and CpOsCp can be directly extended to the pair CpOsB_6_H_11_ and [CpOsBz]^+^. In CpOsB_6_H_11_, osmium again interacts more strongly with the boron ring than with the cyclopentadienyl ligand (−328.2 vs −232.3 kcal mol^–1^; Table S1). The latter interaction is expected to resemble that in [CpOsBz]^+^; however, a direct comparison is not straightforward due to the higher charge of the [OsBz]^2+^ fragment, which leads to significantly larger electrostatic contributions. Despite this difference, the nature of the orbital interactions of Cp^–^ with with [OsB_6_H_11_]^+^ and [OsBz]^2+^ fragments remains remarkably similar in CpOsB_6_H_11_ and [CpOsBz]^+^ (Figure S4), with comparable donor–acceptor interactions governing the metal–ligand bonding.

Let us now return to CpOsCp and the presence of a paratropic ring current around the osmium center, which prevents the formation of the extended diatropic pathway observed in its boron analogue CpOsB_5_H_10_. Osmocene, as a typical sandwich metallocene, is characterized by a relatively rigid structure, limited propensity for structural reorganization, and classical aromatic substitution reactivity localized on the ligands. As we previously demonstrated,^18^ such systems do not exhibit global 3D-aromaticity, but rather localized aromaticity within the Cp rings, a conclusion further supported by the presence of the paratropic current around the metal center. An interesting question is whether this behavior persists across the Group 8 metallocenes.

Ruthenocene displays a similar paratropic ring current around the Ru center; however, in ferrocene, this behavior changes markedly. In this case, the current becomes diatropic, giving rise to an extended diatropic pathway connecting both cyclopentadienyl rings through the iron atom (Figure 6). This transition is accompanied by a strengthening of the aromaticity of the ligands, as reflected in the increase of bond current strengths from 74 (Os) to 75 (Ru) and 81 (Fe). DIAL isosurfaces further support this trend, showing more pronounced paratropic regions around osmium and ruthenium compared to iron. Molecular orbital analysis reveals a greater overlap between the cyclopentadienyl rings in ferrocene, likely due to the shorter inter-ring distance associated with the smaller atomic radius of iron relative to ruthenium and osmium (Figure S5). This enhanced overlap may underlie the observed switch from paratropic behavior in osmocene and ruthenocene to diatropic behavior in ferrocene. Importantly, the above diatropic pathway through Fe should not by itself be interpreted as evidence of global 3D aromaticity. Ferrocene illustrates that metal participation in the induced current may occur without integration of the ligand-centered aromatic systems into a single 3D-aromatic framework. This contrasts with the borane systems, where the metal-involving current is accompanied by extensive metal–borane orbital mixing and substantial changes in the delocalization of the boron framework.

**Figure 6:**
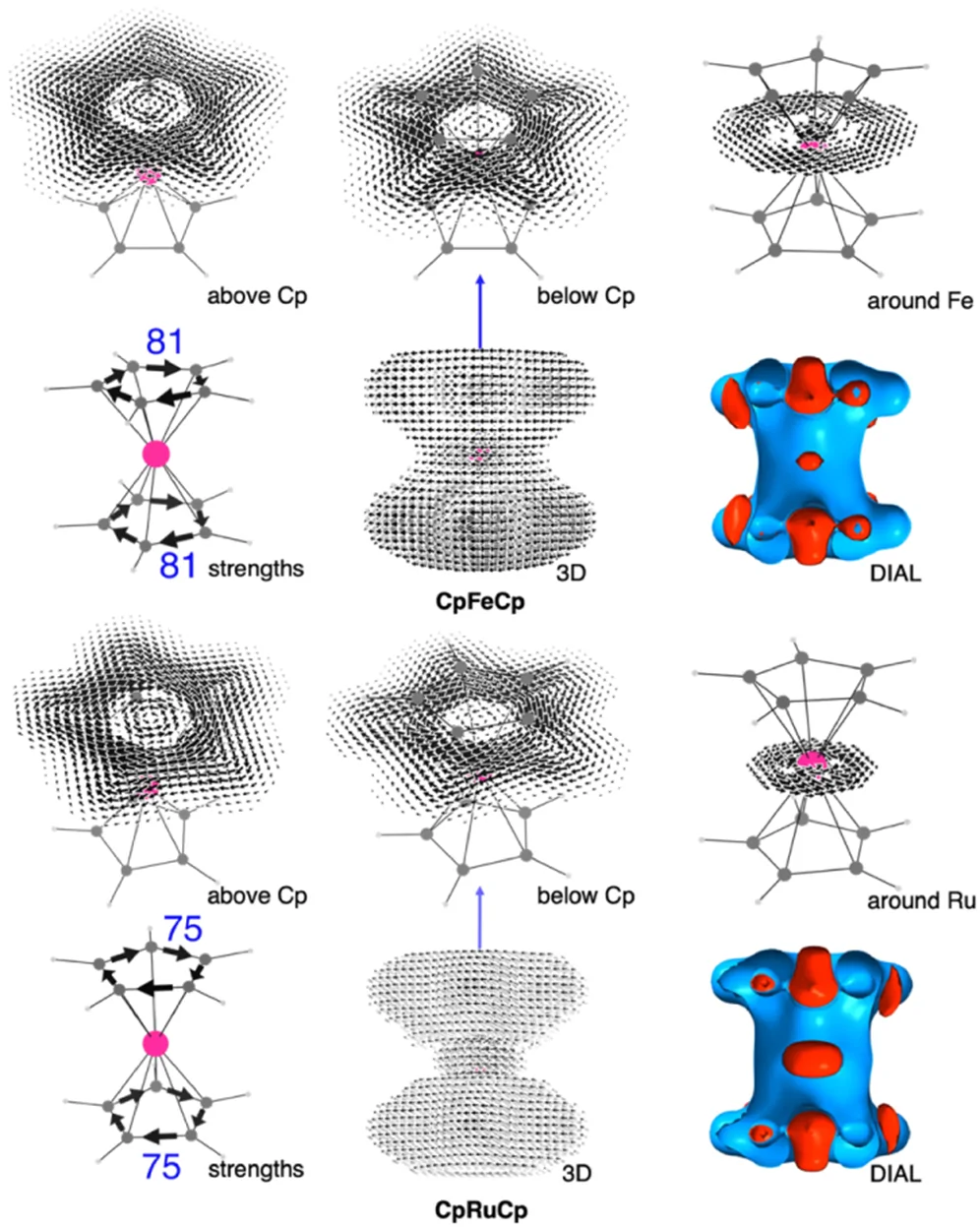
Current density maps of CpFeCp and CpRuCp. Clockwise/anticlockwise circulation corresponds to diatropic/paratropic ring currents. For the ligands, the ring currents are generated at 1 a.u. above and below the ring plane. In the DIAL representation, diatropic regions are shown in blue and paratropic in red. Bond current strengths are given as percentages relative to a reference value of 12 nA T^–1^.

To further quantify this distinction, we compared whole-framework multicenter electron delocalization in ferrocene and its metalloborane analogue [Fe­(B_5_H_10_)_2_], thus keeping the metal center unchanged while replacing the Cp ligands by the corresponding borane frameworks. In ferrocene, the normalized MCI calculated for the C_5_Fe framework amounts to 0.446 au, identical to that obtained for the C_5_ ring alone (0.446 au). Thus, inclusion of the Fe center does not increase the multicenter delocalization measured for the Cp framework. In contrast, for [Fe­(B_5_H_10_)_2_], the MCI increases from 0.271 au for the B_5_ ring to 0.301 au when Fe is included in the multicenter index (B_5_Fe). This difference provides direct electronic evidence that Fe contributes to the multicenter delocalization of the metalloborane framework, whereas the aromatic delocalization of the Cp unit in ferrocene remains predominantly ligand-centered.

This distinction is further supported by a direct comparison of the current-density maps of CpFeCp and [Fe­(B_5_H_10_)_2_] (Figure S10). Although both systems display a metal-involving diatropic pathway, the current density in [Fe­(B_5_H_10_)_2_] is more extensively coupled to the Fe center. This is particularly evident from the 3D current-density and DIAL isosurfaces, which display a substantially broader current-density region around Fe in the metalloborane, whereas a considerably narrower connection through the metal is observed in ferrocene. The current-density maps below the respective ligand planes show the same trend. Therefore, metal participation in a diatropic pathway should not, by itself, be regarded as evidence of 3D aromaticity. Rather, the combined MCI and current-density analyses indicate a substantially greater integration of the metal into the multicenter delocalized framework of [Fe­(B_5_H_10_)_2_] than in ferrocene.

Furthermore, the computed smaller HOMO–LUMO gap in ferrocene relative to ruthenocene and osmocene can be attributed to the more contracted 3d orbitals of iron, which enable more effective overlap with the π orbitals of the cyclopentadienyl ligands. In contrast, the more diffuse 4d and 5d orbitals of ruthenium and osmium result in less efficient orbital overlap and consequently a larger separation between the frontier molecular orbitals (Figure S5).

Finally, although NICS values also suggest an increase in aromaticity for ferrocene, electron-delocalization-based MCI descriptor indicates that the intrinsic aromaticity of the cyclopentadienyl ligands remains essentially constant across the three metallocenes (Figure 7), reinforcing the need for a cautious interpretation of magnetic criteria in such systems.

**Figure 7:**
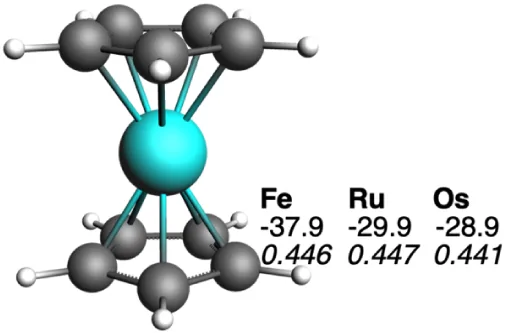
NICS (top value, in ppm) and MCI^1/N^ (bottom value, in a.u., in italics) of Cp rings of CpMCp with M = Fe, Ru and Os.

The above Fe–Ru–Os comparison further shows that the magnetic response involving the metal can vary substantially even within a single transition-metal group. Nevertheless, the electron-delocalization-based MCI of the Cp ligands remains essentially unchanged across the three metallocenes, supporting their predominantly ligand-centered aromatic character within the present series. We emphasize, however, that these results should not be extrapolated to all transition metals. Changes in metal group, oxidation state, electron count, and metal–ligand orbital matching may alter the balance between ligand-localized and metal-integrated delocalization.

At this point, it is worth highlighting the very recent achievement by Jemmis and Ghosh, who have just reported in Science the synthesis and crystallographic characterization of [Os­(B_5_H_10_)_2_].^26^ This compound can be viewed as a carbon-free analogue of ferrocene, but with osmium, and NICS calculations indicate that the aromaticity of its B_5_H_10_ ligands is comparable to that of the cyclopentadienyl rings in ferrocene. Closely related to this finding, and reported even more recently, Merino and Tiznado and coworkers have proposed,^15^ based on quantum chemical calculations, the iron analogue η^5^,η^5^-Fe­(B_5_H_10_)_2_, which does include iron as ferrocene. In view of the close relationship of these compounds to the systems investigated here, we considered it important to include a discussion of both species in the present manuscript. In particular, these authors assign six delocalized 11-center–2-electron orbitals extending across the entire sandwich complex, attributing the resulting electronic delocalization to the boron ligands. Furthermore, the computed magnetically induced current density reveals a diatropic ring current on the B_5_ ring comparable in strength to that of benzene, while a weaker paratropic current is localized closer to the metal center.

First, computed NICS for [Os­(B_5_H_10_)_2_] is somehow smaller in absolute value compared to that for CpOsB_5_H_10_ above (−27.5 vs −35.6 ppm, respectively, Figure 8). Such decrease is further supported by the weaker bond current strengths (49 vs 59, respectively, Figure 9). Conversely, the electronic-based aromaticity criterion MCI assigns a stronger aromaticity to this B_5_H_10_ ring (0.262 au) than that of CpOsB_5_H_10_ (0.225 au). When going up in Group 8, Ru gives almost the same values, whereas [Fe­(B_5_H_10_)_2_] compound appears to present larger aromaticity based on NICS (−33.5 ppm). Current density maps show the stronger diatropic ring current around Fe atom, compared to either Ru or Os that present a partial paratropic ring current, further observed in the corresponding DIAL isosurfaces (Figure 9).

**Figure 8:**
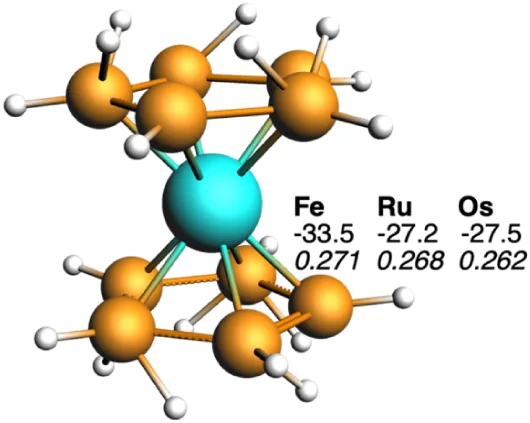
NICS (top value, in ppm) and MCI^1/N^ (bottom value, in a.u., in italics) of B_5_ rings of [M­(B_5_H_10_)_2_] with M = Fe, Ru and Os.

**Figure 9:**
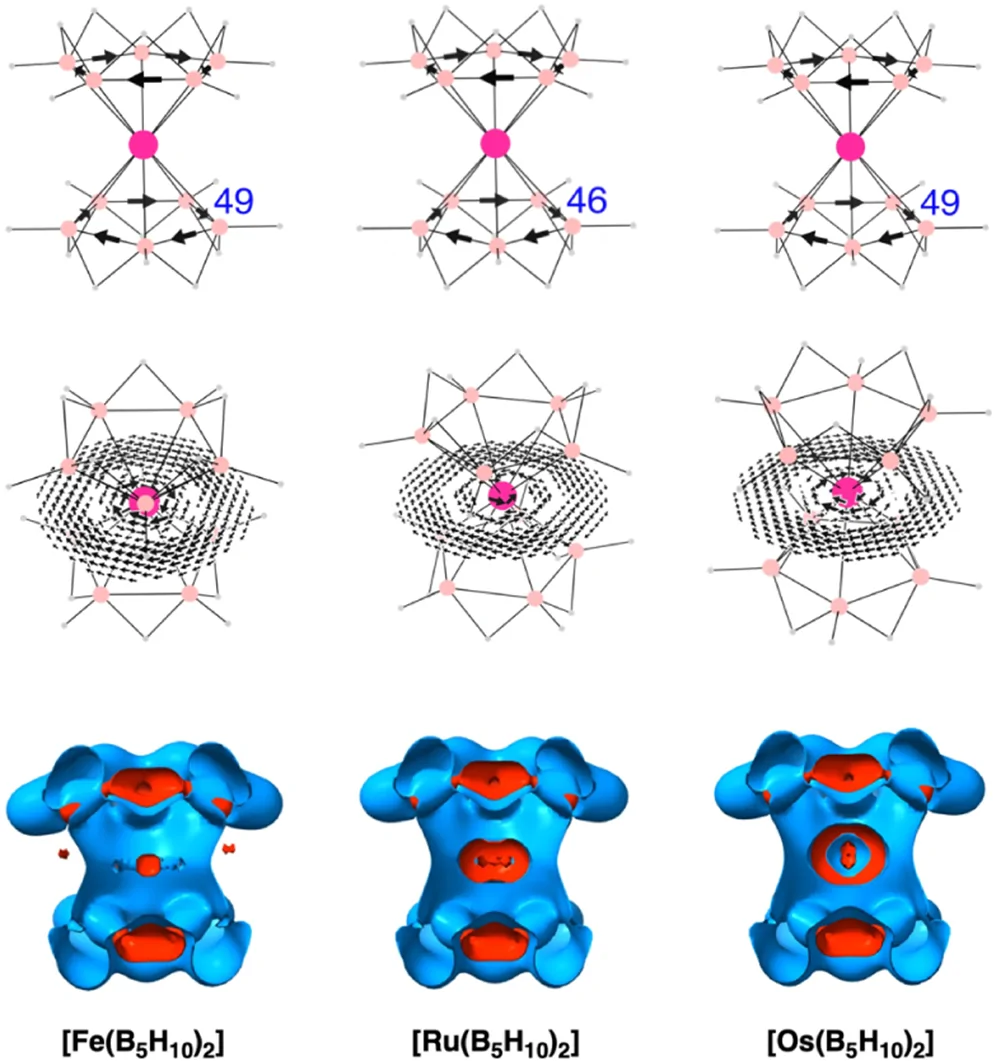
Comparison of current density maps of [M­(B_5_H_10_)_2_] systems with Fe, Os, and Ru. Clockwise/anticlockwise circulation corresponds to diatropic/paratropic ring currents. In the DIAL representation, diatropic regions are shown in blue and paratropic in red. Bond current strengths are given as percentages relative to a reference value of 12 nA T^–1^.

Most importantly, despite the apparent structural similarity between [Os­(B_5_H_10_)_2_] and ferrocene, our results indicate markedly different degrees and topologies of metal involvement in their aromatic delocalization. Within the working classification adopted here, ferrocene is best described as predominantly ligand-centered (2D/2D),^18,27,28^ whereas [Os­(B_5_H_10_)_2_] displays the characteristics of a metal-integrated 3D/3D aromatic framework. Therefore, although the successful synthesis of a carbon-free analogue of ferrocene is highly significant, this analogy should not be extended to their aromatic behavior. At this point, it is worth noting that the [B_5_H_10_]^−^ ligand in [Os­(B_5_H_10_)_2_] is identical to that in CpOsB_5_H_10_ discussed above. Thus, in both complexes, the observed 3D aromaticity originates entirely from the electronic effects of the metal–ligand interaction rather than from any intrinsic structural difference in the ligand itself.

Finally, it should be noted that the high susceptibility of ferrocene toward electrophilic substitution does not necessarily imply a correspondingly larger intrinsic aromaticity of its Cp rings. Reactivity is determined by the complete reaction energy profile and may be strongly influenced by metal-mediated stabilization of intermediates and transition states. Accordingly, the exceptionally facile electrophilic substitution of ferrocene should not be regarded as a direct quantitative aromaticity criterion. In the present analysis, the somewhat stronger diatropic current in ferrocene is accompanied by an essentially unchanged electron-delocalization-based MCI of the Cp rings, illustrating once again that different manifestations of aromaticity need not vary in parallel.

Furthermore, a fundamental distinction between the systems investigated here lies in the electronic consequences of metal coordination. In the metallocenes, the Cp framework largely retains its ligand-centered aromatic delocalization upon coordination, even though the metal may participate in the magnetically induced current pathway. In the metalloborane clusters, by contrast, the metal constitutes an integral vertex of the cluster and its incorporation produces a more extensive reorganization of the electronic delocalization, integrating the metal and borane framework into a multicenter delocalized system. Thus, the relevant distinction is not whether the isolated fragments can exist independently, but the extent to which metal coordination incorporates them into the delocalized framework of the resulting complex.

Last but not least, we have also analyzed a series of 14-vertex bimetallic carboranes inspired by the initial synthesis by Welch and coworkers (Figure 10).^11^ It may seem that this *closo* compound BzRu­(C_2_B_10_H_12_)­RuBz, which contains 14 atoms in its cluster, has little in common with the *nido* compound CpOsB_6_H_11_ discussed above, which consists of 7 atoms in its framework. However, in both cases the osmium (Os) is situated in between a hydrocarbon and a perfectly planar 6-atom B/C ring, despite one is planar B_6_H_11_ and the other three-dimensional C_2_B_10_H_12_. This structural and electronic difference is not as significant when considering the aromaticity indicators NICS and MCI for the boron ring with 6 links. These values are surprisingly similar in both cases, with NICS values of −37.9 and −37.4 ppm, and MCI values of 0.269 and 0.259 au for the *closo* and *nido* compounds, respectively (Figure 10). The structural differences do not correspond to significant differences in these indicators. Additionally, another structural parameter reinforces this similarity: the sum of the distances of the CB_5_ ring in the *closo* compound and the B_6_ ring in the *nido* compound are very similar, measuring 10.532 and 10.538 Å, respectively (Figure 10). This similarity occurs despite the significant differences in substituents in each case, as well as the *closo* or *nido* nature of the compounds and their differing sizes; even the composition of the rings differs, with one being CB_5_ and the other B_6_. All evidence suggests that this is due to the same phenomenon, indicating that both exhibit 3D aromaticity rather than one exhibiting 3D and the other 2D.

**Figure 10:**
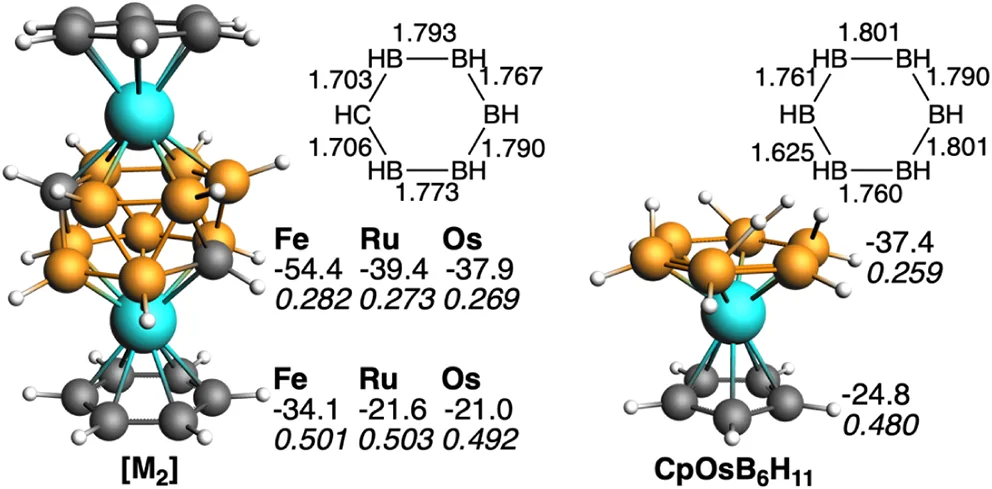
NICS (top value, in ppm) and MCI^1/N^ (bottom value, in a.u., in italics) of C_6_H_6_ and B_5_C rings of Welch’s systems with Fe, Ru and Os and compared to CpOsB_6_H_11_. Bond lengths (in Å) of CB_5_ and B_6_ rings when interacting with osmium are also included.

Further insight into the aromaticity of these compounds is obtained with the current density maps. Again, iron induces a slightly greater increase in the diatropicity of the coordinated benzene ligand, with bond current strengths reaching 122, compared to the corresponding bimetallic ruthenium and osmium complexes, which exhibit values of 116 and 113, respectively (Figures S6 and S7). And the same applies for the CB_5_ rings. It is also observed the larger paratropic contribution by these metals when going down the periodic table given by DIAL isosurfaces (Figures S6 and S7). With respect to this CB_5_ ring, for comparison, in the reference system [B_12_H_12_]^2–^, the bond current strengths of the B_5_ amount to 53, or in the CB_4_ ring of C_2_B_10_H_12_ the bond current strengths are between 46 and 58 (Figure S8).^29^ Such much weaker values in these reference systems further justify the claimed artificial aromaticity increase due to the coupling of the magnetic fields generated by the ligand and the metal atoms. A coupling that is also supported by the MCI for these systems, indicating a decrease in the aromaticity when the benzene ring is coordinated (0.501, 0.503, and 0.492 au for Fe, Ru and Os, respectively).

Finally, to further explore the relationship between the six-membered rings in the *nido* and *closo* structures, we examined the effect of their geometric preorganization (Figures S9 and S10). In both the metallacarborane and all-boron series, formation from the preorganized planar precursor is substantially more favorable than from the corresponding nonpreorganized structure (Δ*G* = −107.2 vs −63.4 kcal mol^–1^ and −121.9 vs −55.4 kcal mol^–1^, respectively). Although these energetic comparisons cannot be regarded as an independent measure of aromaticity, they support a close geometric and electronic relationship between the corresponding *nido* and *closo* frameworks, consistent with the similarities identified by the other electronic and magnetic analyses. The fully boron B_12_H_12_-based systems display the same qualitative trend (Figure S10).

## Conclusions

As a whole, for the systems investigated here, a working classification based on the dominant topology of aromatic delocalization emerges. Systems containing two *nido*-boron frameworks, such as [Os­(B_5_H_10_)_2_], display the characteristics of metal-integrated 3D/3D aromaticity, whereas replacement of one boron framework by a hydrocarbon aromatic ligand gives rise to a mixed regime, exemplified by CpOsB_6_H_11_, in which predominantly ligand-centered 2D aromaticity and metal-integrated 3D aromaticity coexist.^22–25,30^ In contrast, the investigated metallocenes are best described as predominantly ligand-centered 2D/2D aromatic systems. Importantly, this assignment does not imply an absence of metal participation, as demonstrated by the metal-involving diatropic pathway in ferrocene, but rather reflects the dominant topology and extent of multicenter delocalization. The direct comparison between CpFeCp and [Fe­(B_5_H_10_)_2_] supports this distinction, showing that inclusion of Fe increases the multicenter delocalization of the B_5_ framework but not that of the Cp framework.

For the systems investigated here, our results reveal a fundamentally different role of the metal in its interaction with conventional hydrocarbon π ligands and planar borane frameworks, although they may exhibit similar structures in accordance with Wade’s rules and the ECSA. This is likely what prompted Jemmis and Ghosh to use the term bis-*nido*-[Cp*Os­(η^6^-B_6_H_11_)], suggesting that ferrocene may also be classified as a bis-*nido*, although it is commonly referred to as a sandwich compound. Although the Group 8 series considered provides a consistent picture from Fe to Ru and Os, extension of these conclusions to other transition-metal groups will require further investigation.

In the case of sandwich structures, the aromaticity of the arene, especially regarding its most chemical expression—reactivity—behaves very similarly to that of the arene in its isolated state, meaning when it is not coordinated to a metal. This is not the case for a planar borane or carborane bonded to a metal. In a sandwich, the arene exhibits local aromaticity, while the metal-bound planar borane demonstrates global aromaticity. This distinction is fundamental when visualizing the two behaviors: the sandwich exhibits 2D aromaticity, whereas the *nido*-type structure has 3D aromaticity.

Regarding structural aspects, it is not uncommon to find planar six-membered rings capped with a metal in systems exhibiting 3D aromaticity. For instance, we have identified 14-vertex bimetallic carboranes, where the NICS values are notably negative and significantly exaggerated, ranging from −38 to −54 ppm for the Os–Ru–Fe triad, attributed to the influence of the metal. Therefore, it is anticipated that *nido* compounds such as bis-*nido*-[CpOs­(η^6^-B_6_H_11_)] (where Cp = [η^5^-C_5_Me_5_]), will also exhibit negative NICS values.

While metallocene compounds and metallanidoboranes or metallanidocarboranes may occasionally present similar geometries, this work clarifies that the primary distinction between them lies in the nature of the ligands rather than the metal itself. In metallocenes, or sandwich compounds, the geometry of the ligand closely resembles that of its free state. In contrast, within a *nido* structure, the geometry of the nonmetal components significantly alters upon interaction with the metal compared to their configurations in the free state. In the former case, the ligand fragment retains considerable independence from the metal; however, in the latter scenario, the metal exerts a profound influence on the behavior of the carborane or borane fragment, resulting in a situation where these components no longer function as distinct entities. Instead, they merge into a single species characterized by distinctly different properties.

As a whole, based on our findings, we propose a 4-fold criterion to identify 3D-aromaticity: (i) a substantial reorganization of electron delocalization upon coordination, involving extension from the ligand into the metal–ligand framework; (ii) extensive orbital mixing between the metal and ligands; (iii) the presence of distinct diatropic ring currents encompassing the metal center; and (iv) electronic integration of the metal vertex into a globally delocalized three-dimensional framework, rather than preservation of aromaticity predominantly within the individual ligand. Importantly, this latter criterion does not require the corresponding free ligand to be nonplanar or devoid of intrinsic global delocalization. Accordingly, a genuine 3D/3D aromatic system is achieved when both units of the sandwich participate with the metal in 3D aromatic frameworks, as occurs when both units are boron clusters.

## Experimental Section

All calculations were carried out using the AMS package at the ZORA-BLYP-D3­(BJ)/TZ2P level of theory, in toluene (COSMO).^31–34^ Geometry optimizations were performed without symmetry constraints, and analytical Hessians were computed to confirm all stationary points as true minima, as indicated by the absence of imaginary frequencies. Aromaticity was initially assessed using the nucleus-independent chemical shift (NICS) as a magnetic criterion of aromaticity,^35^ and using the gauge-including atomic orbital (GIAO) method.^36^ NICS is defined as the negative value of the absolute magnetic shielding calculated at the center of a ring or at a selected point in space, with large negative values typically associated with aromatic systems. In addition, multicenter indices (MCI)^37–39^ were computed to further quantify electron delocalization and assess aromatic character, at the same ZORA-BLYP-D3­(BJ)/TZ2P level with ADF.

Current density maps were computed using the SYSMOIC program at the B3LYP-GD3/def2-TZVP//ZORA-BLYP-D3­(BJ)/TZ2P level,^40–45^ applying a perpendicular magnetic field over a plane located 1 au below the molecular plane. Pseudopotentials (SDD ECP) have been included to correctly treat the effect of the core electrons on the computed ring current of Fe, Ru, and Os atoms.^46^ Red and blue arrows indicate regions where the component of the current density parallel or antiparallel to the magnetic field exceeds 30% of the total vector magnitude, with diatropic and paratropic circulations corresponding to clockwise and anticlockwise directions, respectively.^47^ These maps allow differentiation between σ and π contributions: σ-electrons generate localized vortices, whereas π-electrons produce global ring currents responsible for shielding inside and deshielding outside the ring. At ∼1 au from the ring plane, the current density is dominated by π-electrons, whose delocalized circulation masks the weaker σ contributions. Nonuniform current patterns in a ring indicate weak aromaticity. Quantitative assessment was achieved through bond current strengths expressed in percentage relative to benzene, which has a bond current strength of 12 nA T^–1^.^40–42,47–49^ Additionally, the magnetically induced isotropically averaged Lorentz force density (DIAL) was evaluated as a complementary real-space descriptor.^47,48^ Derived from the induced current density, DIAL identifies regions of current reinforcement (diamagnetic) and depletion (paramagnetic), providing an orientation-independent picture of electron delocalization. In the graphical representations, diatropic regions are shown in blue and paratropic regions in red, using isovalues of −0.001 and +0.006 au, respectively. To check the effect of the functional, current density maps for both CpOsB_6_H_11_ and CpOsB_5_H_10_ systems have also been computed at the PBE0-GD3/def2-TZVP level of theory. For CpOsB_6_H_11_, bond current strengths are reduced to 66 and 61 nA T^–1^ for the boron and carbon rings, respectively; whereas those for CpOsB_5_H_10_ are reduced to 54 and 61 nA T^–1^ for the boron and carbon rings, respectively, thus keeping the same trends.

Finally, the interaction between the metal and the boron or carbon rings was analyzed within the framework of quantitative Kohn–Sham molecular orbital theory in combination with a quantitative energy decomposition analysis (EDA) in the gas phase. The interaction energy Δ*E*_int_ was decomposed into the classical electrostatic attraction Δ*V*_elstat_, Pauli repulsion Δ*E*_Pauli_ between occupied orbitals, stabilizing orbital interactions Δ*E*_oi_, and dispersion Δ*E*_disp._ ^32,34^ It is worth noting that point NICS values, particularly those evaluated at ring centers in metal-containing systems, may contain substantial contributions from the magnetic response of the metal that are not directly related to aromaticity. Accordingly, NICS values are not used here as a stand-alone criterion for aromaticity. Aromaticity assignments are instead based on their combined interpretation with current-density maps, MCI values, and orbital/bonding analyses.

## Supplementary Material



## Notes

Complementary current density maps, highest occupied molecular orbitals, energy decomposition analysis, molecular orbitals involved in donor–acceptor interactions, formation energies, and Cartesian coordinates of all computed systems (PDF)

## References

[1] Wade K. (1971). Structural
Significance of Number of Skeletal Bonding Electron-Pairs in Carboranes,
Higher Boranes and Borane Anions, and Various Transition-Metal Carbonyl
Cluster Compounds. J. Chem. Soc. D Chem. Commun..

[2] Wade K. (2009). Bonding with
boron. Nat. Chem..

[3] Williams R. E. (1971). Carboranes
and boranes; polyhedra and polyhedral fragments. Inorg. Chem..

[4] Rudolph R.
W., Pretzer W. R. (1972). Hueckel-type
rules and the systematization of borane
and heteroborane chemistry. Inorg. Chem..

[5] Kar K., Joshi G., Jemmis E. D., Ghosh S. (2026). All-boron analogue
of planar benzene on an osmium template. Chem.
Sci..

[6] Alexandrova A. N., Birch K. A., Boldyrev A. I. (2003). Flattening the B_6_H_6_
^2–^ Octahedron. Ab Initio Prediction of a
New Family of Planar All-Boron Aromatic Molecules. J. Am. Chem. Soc..

[7] Weiss R., Grimes R. N. (1977). New ferraboranes. Structural analogs of hexaborane(10)
and ferrocene. A complex of cyclic B_5_H_10_
^–^, a counterpart of C_5_H_5_. J. Am. Chem. Soc..

[8] Weiss R., Grimes R. N. (1979). Polyhedral ferraboranes derived from the B_5_H_8_
^–^ ion. Analogs of ferrocene, hexaborane(10),
and nido-B_11_H_15_. Inorg.
Chem..

[9] Ferrer M., Alkorta I., Elguero J., Barrios-Llacuachaqui J. R., Tiznado W., Oliva-Enrich J. M. (2024). Striking
Borane Planarization in
the Thermal Rearrangement (η^5^-C_5_H_5_)­Fe­(η^3^-B_5_H_10_)→(η^5^-C_5_H_5_)­Fe­(η^5^-B_5_H_10_). Chem. —Eur. J..

[10] Yao H., Sabat M., Grimes R. N. (2002). Wired Multidecker Sandwich Assemblies.
Stepwise Construction of a Hexanuclear Benzene-Centered Tris­(alkynyl
triple-decker) Complex. Organometallics.

[11] Ellis D., Lopez M. E., McIntosh R., Rosair G. M., Welch A. J. (2005). Fourteen-vertex
homo- and heterobimetallic metallacarboranes. Chem. Commun..

[12] Poater J., Solà M., Viñas C., Teixidor F. (2013). A Simple Link between
Hydrocarbon and Borohydride Chemistries. Chem.—Eur.
J..

[13] Poater J., Solà M., Viñas C., Teixidor F. (2014). π Aromaticity
and Three-Dimensional Aromaticity: Two sides of the Same Coin?. Angew. Chem., Int. Ed..

[14] Poater J., Solà M., Viñas C., Teixidor F. (2016). Hückel’s
Rule of Aromaticity Categorizes Aromatic closo Boron Hydride Clusters. Chem. —Eur. J..

[15] Roman-Ventura V., Quispe-Corimayhua L., Sacanamboy D. S., Inostroza D., Leyva-Parra L., Páez D., Donald K. J., Merino G., Tiznado W. (2026). A Borane Sandwich Analogue of Ferrocene. Angew. Chem., Int. Ed..

[16] Efraty A. (1977). Cyclobutadienemetal
complexes. Chem. Rev..

[17] Feixas F., Jiménez-Halla J. O. C., Matito E., Poater J., Solà M. (2007). Is the Aromaticity
of the Benzene Ring in the (η^6^-C_6_H_6_)­Cr­(CO)_3_ complex Larger
than that of the Isolated Benzene Molecule?. Polish. J. Chem..

[18] Poater J., Viñas C., Bennour I., Escayola Gordils S., Solà M., Teixidor F. (2020). Too Persistent to Give Up: Aromaticity
in Boron Clusters Survives Radical Structural Changes. J. Am. Chem. Soc..

[19] El
Bakouri O., Szczepanik D. W., Jorner K., Ayub R., Bultinck P., Solà M., Ottosson H. (2022). Three-Dimensional Fully
π-Conjugated Macrocycles: When 3D-Aromatic and When 2D-Aromatic-in-3D?. J. Am. Chem. Soc..

[20] Noori Z., Poater J. (2025). Aromaticity switch of borabenzene: from aromatic when
free or weakly aromatic when fused to 2D PAHs to non-aromatic when
fused to 3D carboranes. Org. Chem. Front..

[21] Noori Z., Poater J. (2026). Carbon–carbon
to boron–nitrogen isosterism
enables switching toward fused 3D/2D magnetically aromatic systems. Org. Chem. Front..

[22] Noori Z., Solà M., Viñas C., Teixidor F., Poater J. (2025). Unraveling
aromaticity: the dual worlds of pyrazole, pyrazoline, and 3D carborane. Beilstein J. Org. Chem..

[23] Poater J., Viñas C., Solà M., Teixidor F. (2022). 3D and 2D aromatic
units behave like oil and water in the case of benzocarborane derivatives. Nat. Commun..

[24] Buzsaki D., Kovacs M. B., Humpfner E., Harcsa-Pinter Z., Kelemen Z. (2022). Conjugation between 3D and 2D aromaticity:
does it
really exist? The case of carborane-fused heterocycles. Chem. Sci..

[25] Buzsáki D., Gál D., Harcsa-Pintér Z., Kalabay L., Kelemen Z. (2024). The possible aromatic conjugation via the different
edges of (car)­borane clusters: Can the relationship between 3D and
2D aromatic systems be reconciled?. Chem. —Eur.
J..

[26] Mohapatra S., Saha S., Joshi G., Gupta U., Patel D. K., Jemmis E. D., Ghosh S. (2026). [Os­(η^5^-B_5_H_10_)_2_]: A carbon-free analog
of ferrocene. Science.

[27] Bean D. E., Fowler P. W., Morris M. J. (2011). Aromaticity
and ring currents in
ferrocene and two isomeric sandwich complexes. J. Organomet. Chem..

[28] Phillips L., Separovic F., Aroney M. J. (2003). The aromaticity of ferrocene and
some derivatives, ruthenocene and dibenzenechromium as determined
via ring current assessment and ^13^C anisotropic contributions
to the ^1^H NMR shielding. New J. Chem..

[29] Poater J., Viñas C., Olid D., Solà M., Teixidor F. (2022). Aromaticity and Extrusion
of Benzenoids Linked to [*o*-COSAN]^–^: Clar Has the Answer. Angew. Chem., Int. Ed..

[30] Gál D., Szántai L., Buzsáki D., Kelemen Z. (2025). Unveiling the True
Identity of Carborane-Fused Phosphorus Heterocycles. Org. Lett..

[31] te
Velde G., Bickelhaupt F. M., Baerends E. J., Fonseca
Guerra C., van Gisbergen S. J. A., Snijders J. G., Ziegler T. (2001). Chemistry
with ADF. J. Comput. Chem..

[32] Bickelhaupt, F. M. ; Baerends, E. J. Kohn-Sham Density Functional Theory: Predicting and Understanding Chemistry. In Reviews in Computational Chemistry, Lipkowitz, K. B. ; Boyd, D. B. 15; Wiley-VCH: New York, 2000; 1–86.

[33] Vermeeren P., Hamlin T. A., Bickelhaupt F. M. (2021). Chemical
reactivity from an activation
strain perspective. Chem. Commun..

[34] Vermeeren P., van der Lubbe S. C. C., Fonseca Guerra C., Bickelhaupt F. M., Hamlin T. A. (2020). Understanding chemical reactivity using the activation
strain model. Nature Protoc..

[35] Chen Z. F., Wannere C. S., Corminboeuf C., Puchta R., Schleyer P. v.R. (2005). Nucleus-independent
chemical shifts (NICS) as an aromaticity criterion. Chem. Rev..

[36] Wolinski K., Hinton J. F., Pulay P. (1990). Efficient Implementation of the Gauge-Independent
Atomic Orbital Method for NMR Chemical-Shift Calculations. J. Am. Chem. Soc..

[37] Bultinck P., Ponec R., Van Damme S. (2005). Multicenter bond indices as a new
measure of aromaticity in polycyclic aromatic hydrocarbons. J. Phys. Org. Chem..

[38] Feixas F., Matito E., Poater J., Solà M. (2015). Quantifying
aromaticity with electron delocalisation measures. Chem. Soc. Rev..

[39] Matito E., Duran M., Solà M. (2005). The aromatic fluctuation index (FLU):
A new aromaticity index based on electron delocalization. J. Chem. Phys..

[40] Monaco G., Summa F. F., Zanasi R. (2021). Program Package
for the Calculation
of Origin-Independent Electron Current Density and Derived Magnetic
Properties in Molecular Systems. J. Chem. Inf.
Model..

[41] Monaco G., Summa F. F., Zanasi R. (2020). Atomic size adjusted calculation
of the magnetically induced current density. Chem. Phys. Lett..

[42] Berger R. J. F., Monaco G., Zanasi R. (2020). On the topology of total and diamagnetic
induced electronic currents in molecules. J.
Chem. Phys..

[43] Becke A. D. (1993). Density-Functional
Thermochemistry. III. The Role of Exact Exchange. J. Chem. Phys..

[44] Lee C. T., Yang W. T., Parr R. G. (1988). Development of the Colle-Salvetti
Correlation-Energy Formula into a Functional of the Electron-Density. Phys. Rev. B.

[45] Stephens P. J., Devlin F. J., Chabalowski C. F., Frisch M. J. (1994). Ab-Initio Calculation
of Vibrational Absorption and Circular-Dichroism Spectra Using Density-Functional
Force-Fields. J. Phys. Chem..

[46] Orozco-Ic M., Soriano-Agueda L., Sundholm D., Matito E., Merino G. (2024). Core-electron
contributions to the magnetic response of molecules with heavy elements
and their significance in aromaticity assessments. Chem. Sci..

[47] Monaco G., Zanasi R. (2020). The molecular electronic structure revealed by the
magnetically induced Lorentz force density. J. Chem. Phys..

[48] Monaco G., Summa F. F., Zanasi R., Lazzeretti P. (2024). Electronic
Current Density Induced by Uniform Magnetic Fields in Clarenes. Chem. —Eur. J..

[49] Orza M., Summa F. F., Zanasi R., Monaco G. (2024). A Study of Differential
Topology on the Magnetically Induced Isotropically Averaged Lorentz
Force Density of a Few Simple Molecules. Molecules.

