# Supplementary material for: Metal-Containing Borane and Hydrocarbon Sandwich Complexes: Exploring the 2D–3D Aromaticity Dilemma
Source: Inorg Chem. 2026 Sep 24;65(40):23833–44. doi: 10.1021/acs.inorgchem.6c03817 (PMC13664074; doi:10.1021/acs.inorgchem.6c03817)
Supplement: Supplementary file 1 [file ic6c03817_si_001.pdf]

SUPPORTING INFORMATION

Metal-Containing Borane and Hydrocarbon Sandwich Complexes: Exploring the 2D-3D Aromaticity Dilemma

Zahra Noori,[1] Clara Viñas,[2] Miquel Solà,[3*] Francesc Teixidor,[2*] and Jordi Poater[1,4*]

[1]Departament de Química Inorgànica i Orgànica & Institut de Química Teòrica i Computacional (IQTCUB), Universitat de Barcelona, 08028 Barcelona, Spain. E-mail: jordi.poater@ub.edu
[2]Institut de Ciència de Materials de Barcelona, Consejo Superior de Investigaciones Científicas, 08193 Bellaterra, Spain. E-mail: teixidor@icmab.es
[3]Institut de Química Computacional i Catàlisi and Departament de Química, Universitat de Girona, 17003 Girona, Catalonia, Spain. E-mail: miquel.sola@udg.edu
[4]ICREA, 08010 Barcelona, Spain

Contents

Figure S1. Current density maps of $[B_6H_{11}]^-$ and $[B_5H_{10}]^-$. Clockwise/anticlockwise circulation corresponds to diatropic/paratropic ring currents (left and middle). In the DIAL representation, diatropic regions are shown in blue and paratropic in red (right). **S2**
Figure S2. Highest occupied molecular orbitals of $CpOsB_6H_{11}$ (left) and $[CpOsBz]^+$ (right) with the contribution from the d/p orbitals of osmium (in percentage) and their energies (in eV). **S3**
Table S1. Energy decomposition analysis (in kcal mol^{-1}) of the boron clusters and their hydrocarbon counterparts. Percentages over all attractive interactions (ΔV_{elstat} + ΔE_{oi} + ΔE_{disp}) are given for ΔV_{elstat} and ΔE_{oi} (values below). **S4**
Figure S3. Molecular orbitals involved in the main donor-acceptor interaction of $CpOsB_5H_{10}$ involving the breaking between the boron ring and the osmium, with the corresponding Gross Mulliken populations included (in electrons). H and L refer to HOMO and LUMO, respectively. **S5**
Figure S4. Molecular orbitals involved in the main donor-acceptor interaction of $CpOsB_6H_{11}$ and $[CpOsBz]^+$ involving the breaking between the osmium and the cyclopentadienyl, with the corresponding Gross Mulliken populations included (in electrons). H and L refer to HOMO and LUMO, respectively. **S6**
Figure S5. Highest occupied molecular orbitals of CpOsCp and CpFeCp with the contribution from the d/p orbitals of osmium and iron (in percentage), and their energies (in eV). **S7**
Figure S6. Current density maps of Welch's systems with Os and Ru. Clockwise/anticlockwise circulation corresponds to diatropic/paratropic ring currents. In the DIAL representation, diatropic regions are shown in blue and paratropic in red. Bond current strengths are given as percentages relative to a reference value of 12 nA T^{-1}. **S8**
Figure S7. Current density maps of Welch's systems with Fe. Clockwise/anticlockwise circulation corresponds to diatropic/paratropic ring currents. In the DIAL representation, diatropic regions are shown in blue and paratropic in red. Bond current strengths are given as percentages relative to a reference value of 12 nA T^{-1}. **S9**
Figure S8. NICS (top value, in ppm), MCI$^{1/N}$ (bottom value, in italics, in a.u.), and bond current strengths (given as percentages relative to a reference value of 12 nA T^{-1}) of reference *closo* clusters $C_2B_{10}H_{12}$ and $[B_{12}H_{12}]^{2-}$. **S10**
Figure S9. Formation energies (in kcal mol^{-1}) of $BzOs(C_2B_{10}H_{12})OsBz$ and $C_2B_{12}H_{14}$. **S11**
Figure S10. Formation energies (in kcal mol^{-1}) of $[BzOs(B_{12}H_{12})OsBz]^{2-}$ and $[B_{14}H_{14}]^{2-}$. **S13**
Figure S11. Current density maps of CpFeCp and $[Fe(B_5H_{10})_2]$. Clockwise/anticlockwise circulation corresponds to diatropic/paratropic ring currents. In the DIAL representation, diatropic regions are shown in blue and paratropic in red. Bond current strengths are given as percentages relative to a reference value of 12 nA T^{-1}. **S14**
Table S2. Cartesian coordinates (in Å) and ADF electronic energies (in kcal mol^{-1}) of all systems under analysis. Computed at ZORA-BLYP-D3(BJ)/TZ2P in toluene (COSMO). **S15**

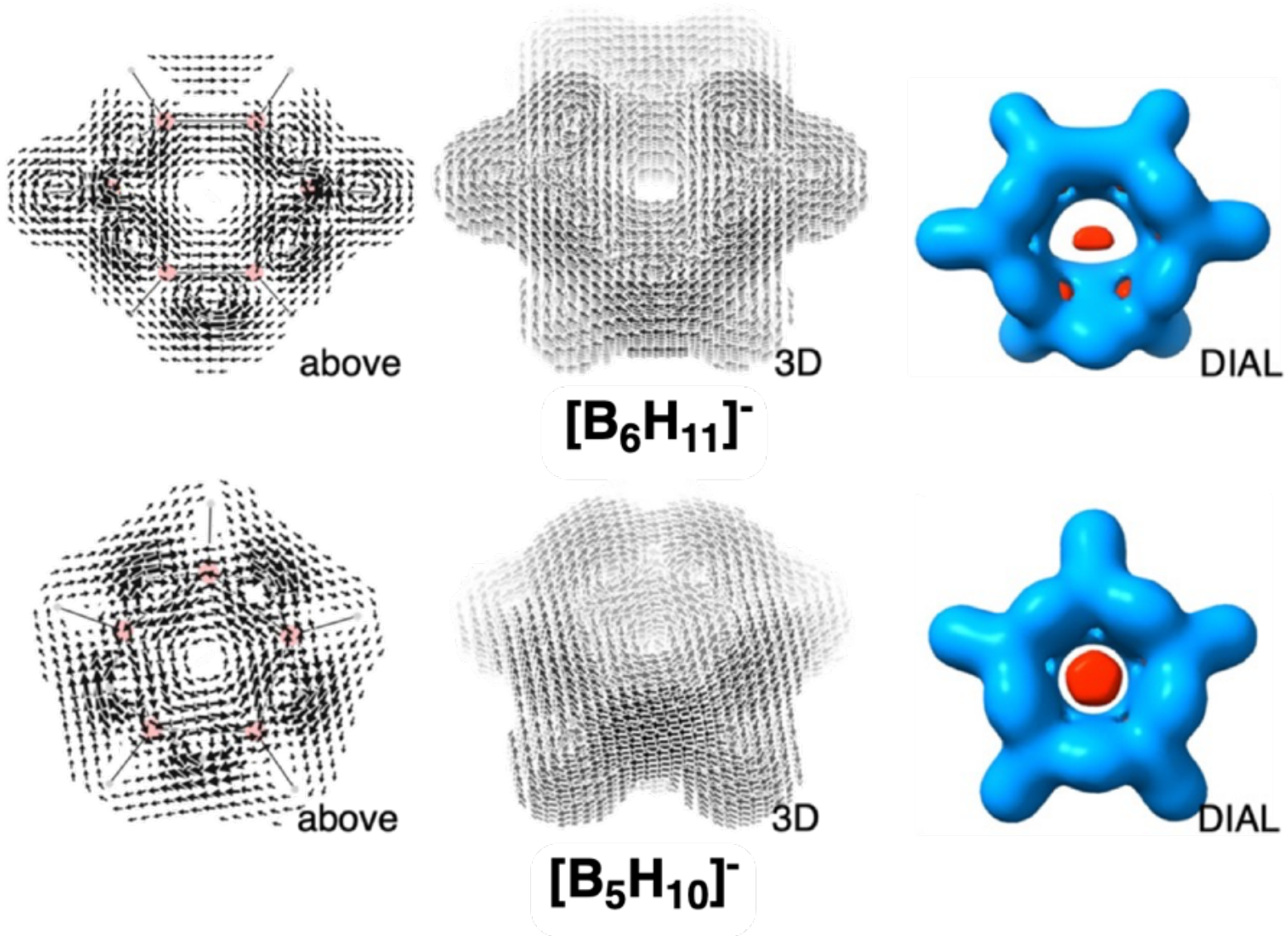

Figure S1. Current density maps of $[B_6H_{11}]^-$ and $[B_5H_{10}]^-$. Clockwise/anticlockwise circulation corresponds to diatropic/paratropic ring currents (left and middle). In the DIAL representation, diatropic regions are shown in blue and paratropic in red (right).

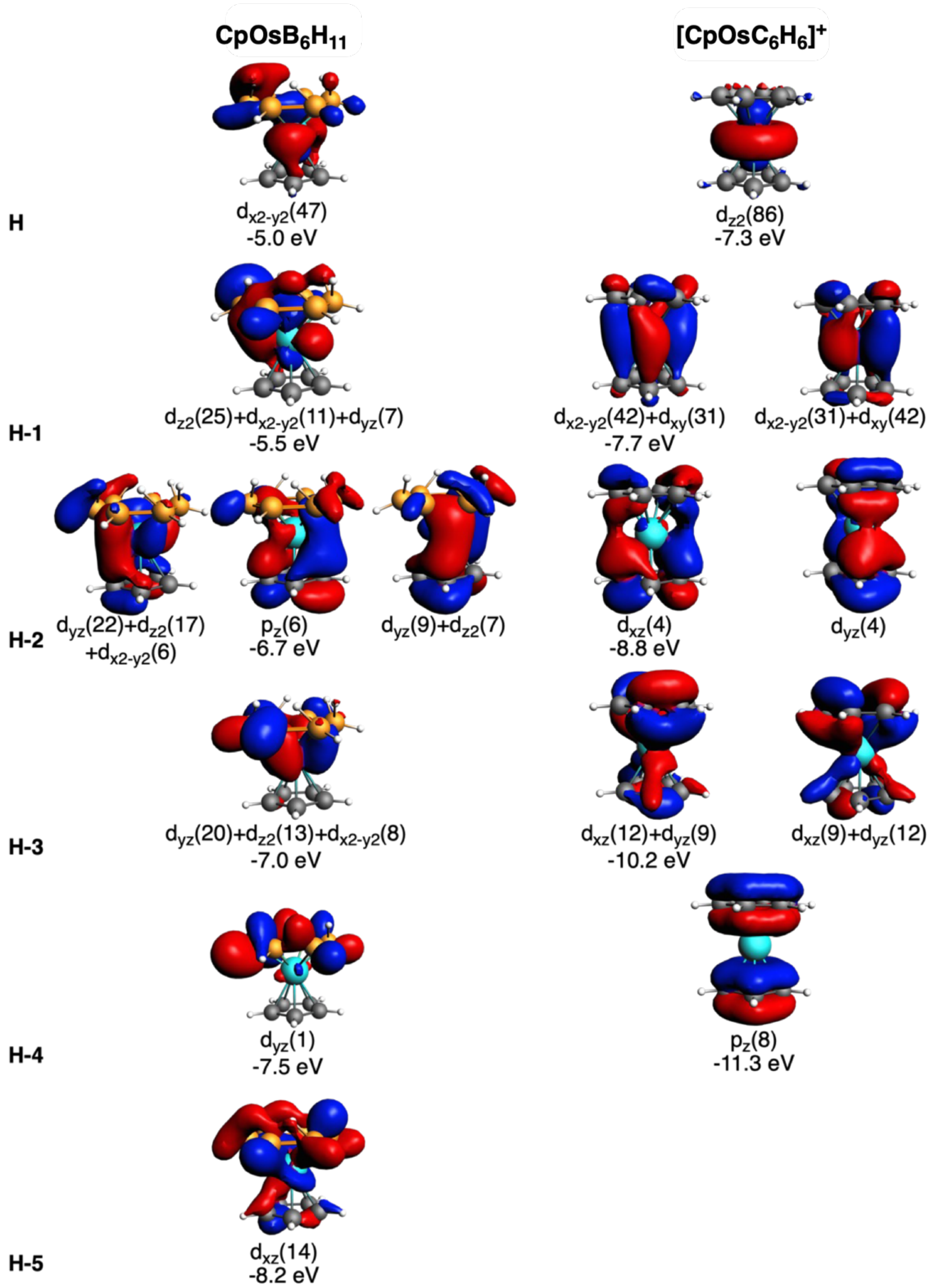

Figure S2. Highest occupied molecular orbitals of $CpOsB_6H_{11}$ (left) and $[CpOsC_6H_6]^+$ (right) with the contribution from the d/p orbitals of osmium (in percentage) and their energies (in eV).

Table S1. Energy decomposition analysis (in kcal mol^{-1}) of the boron clusters and their hydrocarbon counterparts. Percentages over all attractive interactions (ΔV_{elstat} + ΔE_{oi} + ΔE_{disp}) are given for ΔV_{elstat} and ΔE_{oi} (values below).[a,b]

system	fragments	ΔE_{int}	ΔE_{Pauli}	ΔV_{elstat}	ΔE_{oi}	ΔE_{disp}
**$CpOsB_5H_{10}$**	Cp^- + $[OsB_5H_{10}]^+$	-220.9	210.7	-259.1	-163.6	-9.0
				60%	38%	
	$[CpOs]^+$ + $[B_5H_{10}]^-$	-335.0	462.5	-452.0	-331.1	-14.4
				57%	42%	
**CpOsCp**	Cp^- + $[OsCp]^+$	-242.5	259.7	-290.8	-204.2	-7.1
				58%	41%	
**$CpOsB_6H_{11}$**	Cp^- + $[OsB_6H_{11}]^+$	-232.3	256.4	-287.8	-188.3	-12.7
				59%	39%	
	$[CpOs]^+$ + $[B_6H_{11}]^-$	-328.2	445.0	-437.2	-318.6	-17.4
				57%	41%	
**$[CpOsBz]^+$**	Cp^- + $[OsBz]^{2+}$	-389.0	249.9	-373.7	-257.3	-8.0
				58%	40%	
	$[CpOs]^+$ + Bz	-110.8	250.7	-155.5	-197.5	-8.5
				43%	55%	

[a] Computed at ZORA-BLYP-D3(BJ)/TZ2P level of theory. [b] $\Delta E_{int} = \Delta E_{Pauli} + \Delta V_{elstat} + \Delta E_{oi} + \Delta E_{disp}$.

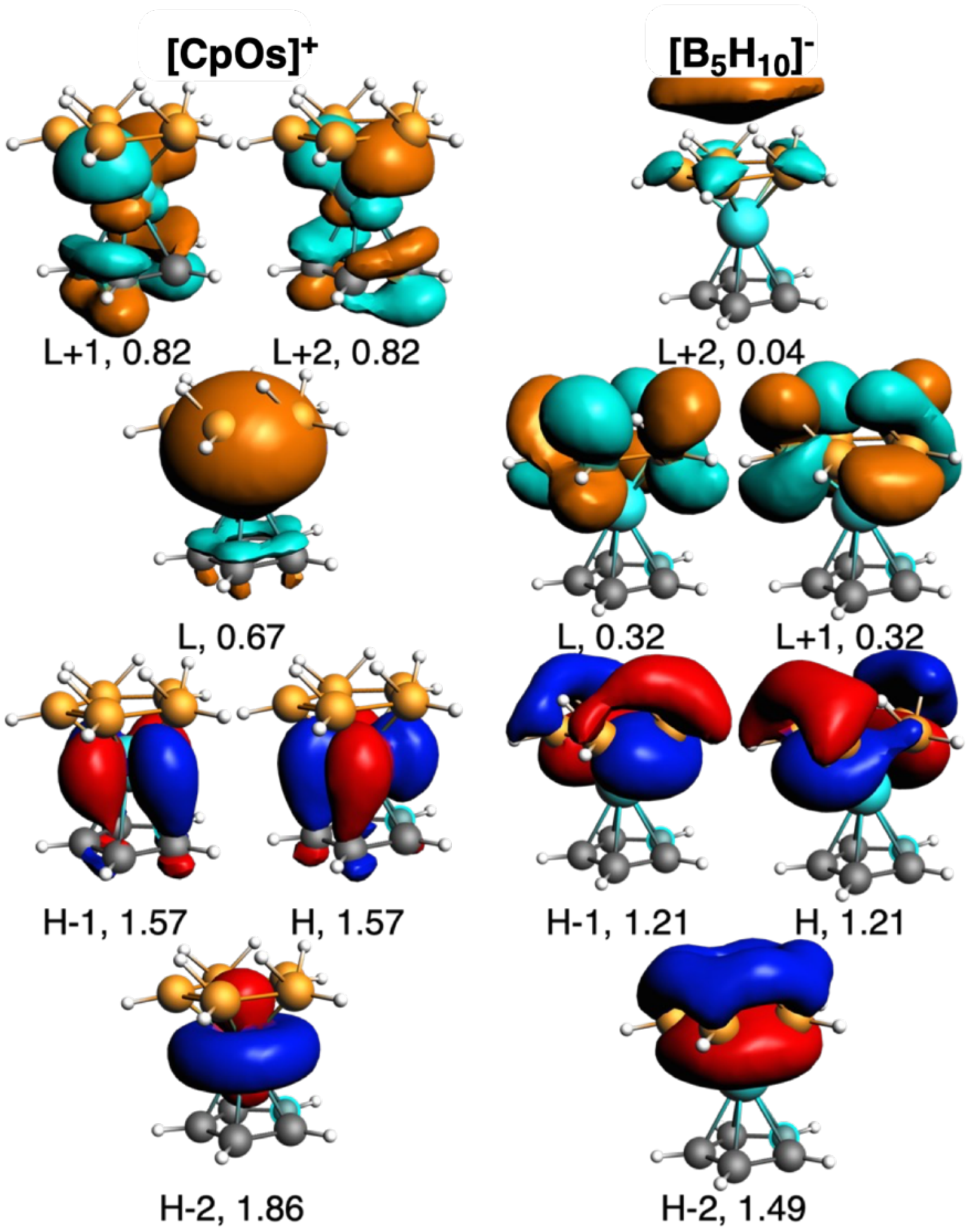

Figure S3. Molecular orbitals involved in the main donor-acceptor interaction of $CpOsB_5H_{10}$ involving the breaking between the boron ring and the osmium, with the corresponding Gross Mulliken populations included (in electrons). H and L refer to HOMO and LUMO, respectively.

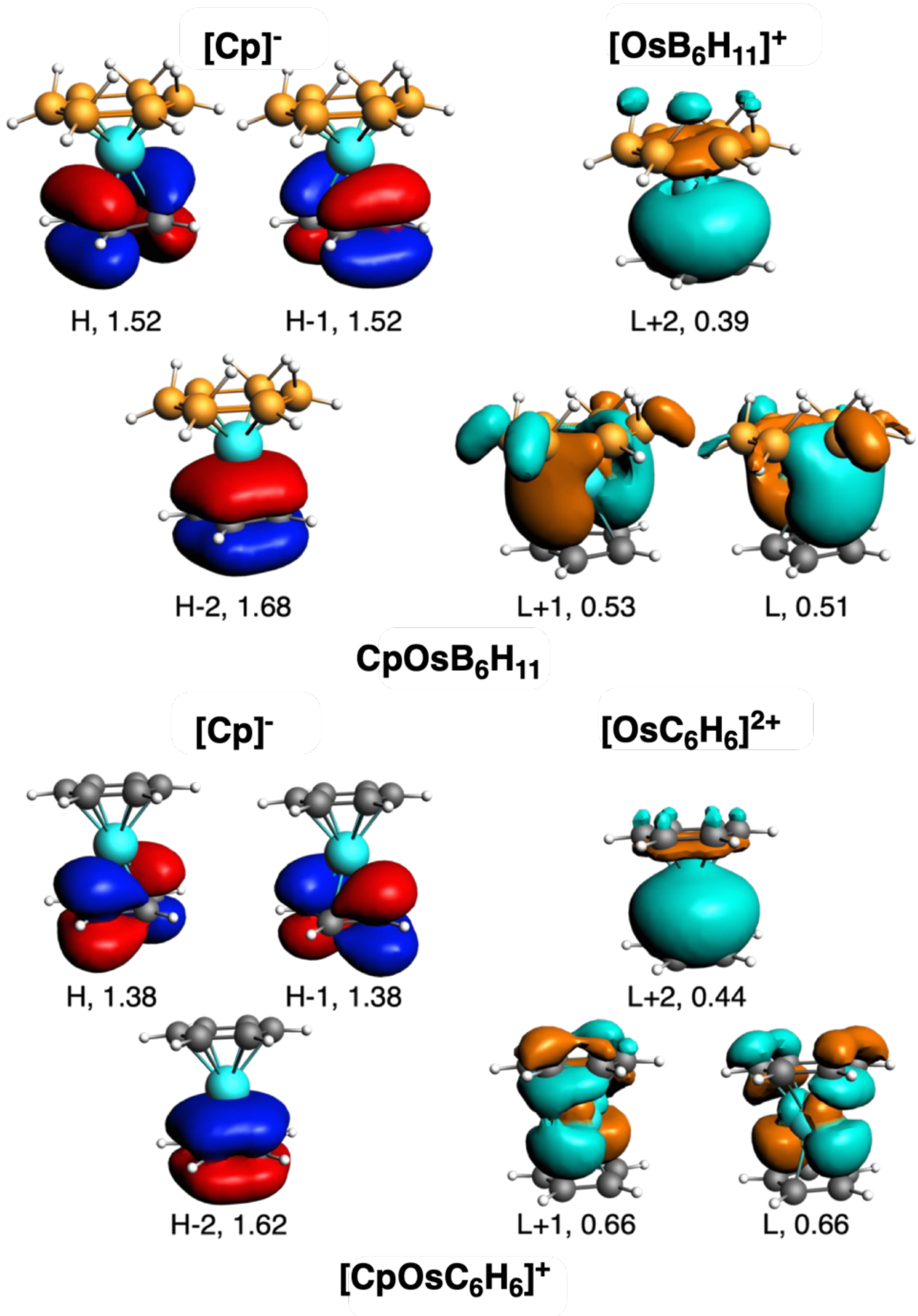

Figure S4. Molecular orbitals involved in the main donor-acceptor interaction of $CpOsB_6H_{11}$ and $[CpOsBz]^+$ involving the breaking between the osmium and the cyclopentadienyl, with the corresponding Gross Mulliken populations included (in electrons). H and L refer to HOMO and LUMO, respectively.

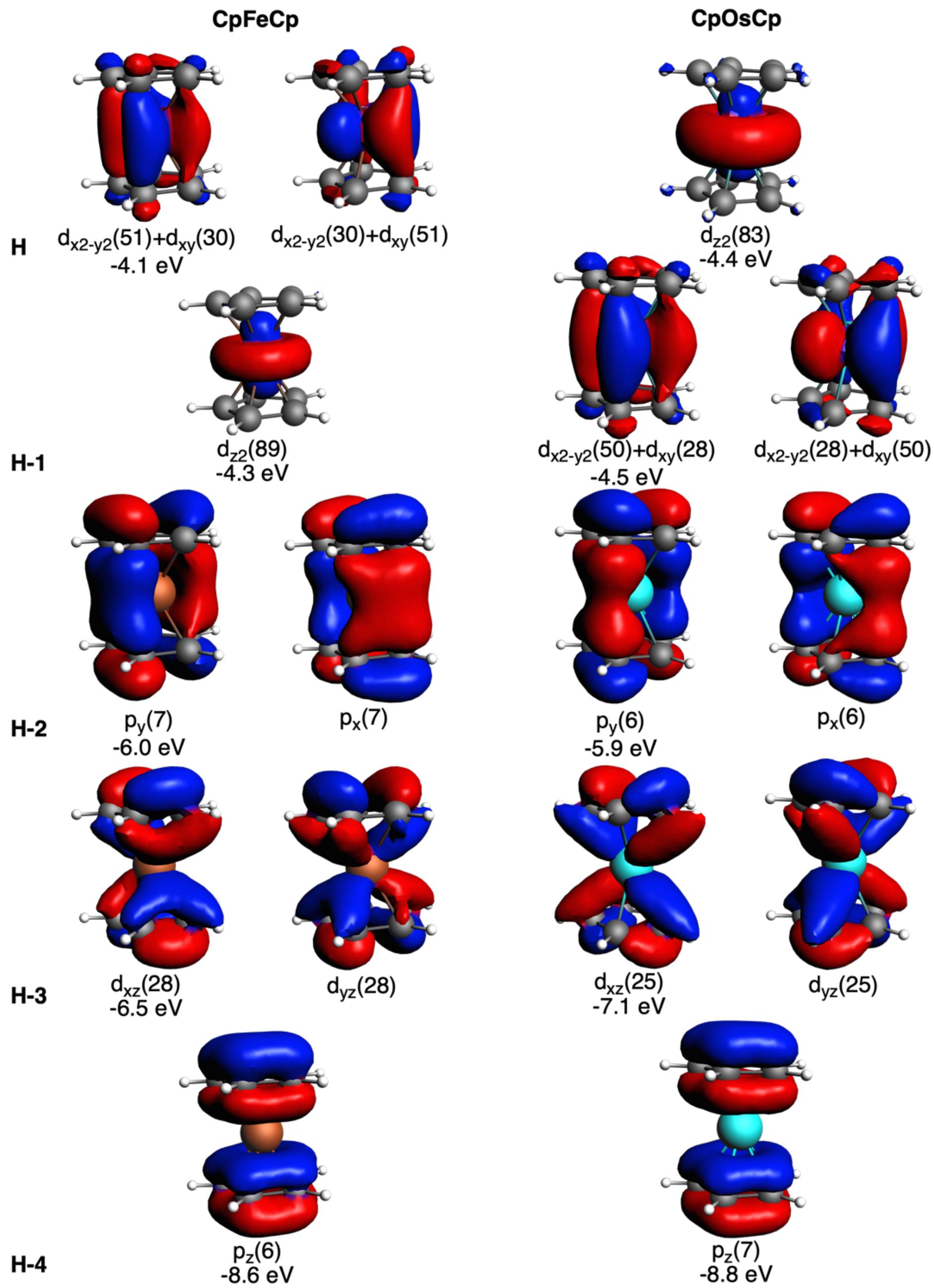

Figure S5. Highest occupied molecular orbitals of CpOsCp and CpFeCp with the contribution from the d/p orbitals of osmium and iron (in percentage), and their energies (in eV).

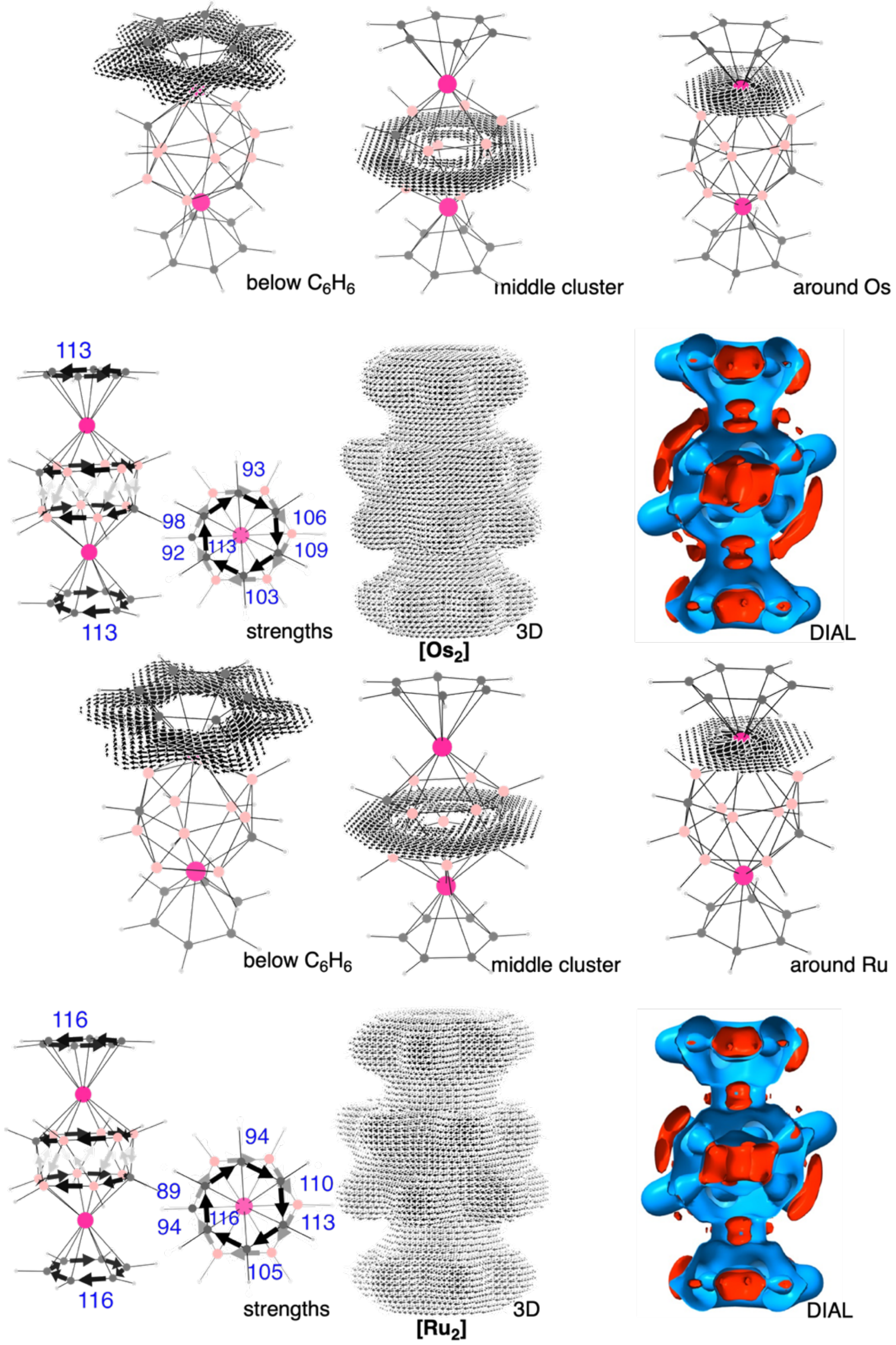

Figure S6. Current density maps of Welch's systems with Os and Ru. Clockwise/anticlockwise circulation corresponds to diatropic/paratropic ring currents. In the DIAL representation, diatropic regions are shown in blue and paratropic in red. Bond current strengths are given as percentages relative to a reference value of 12 nA T^{-1}.

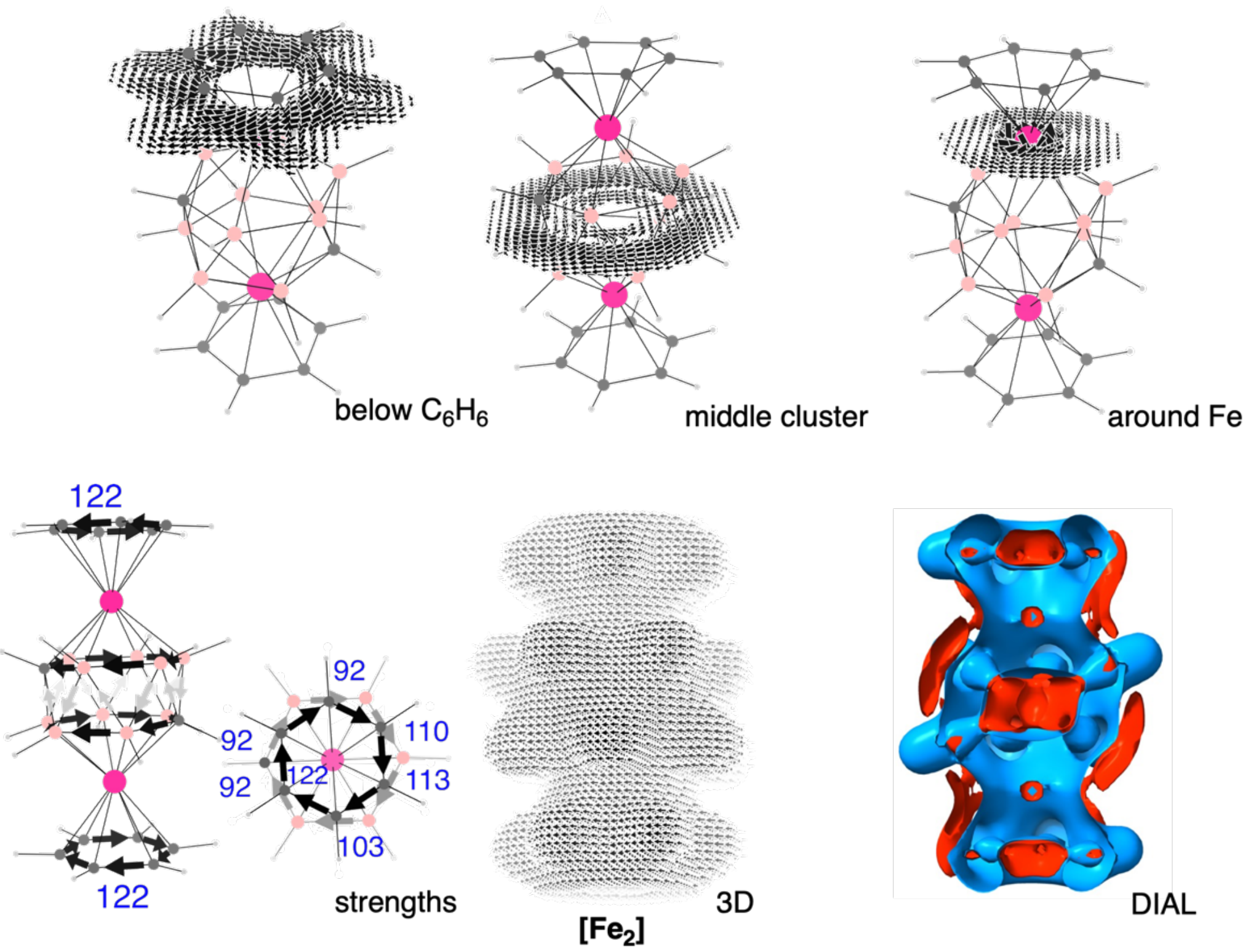

Figure S7. Current density maps of Welch's systems with Fe. Clockwise/anticlockwise circulation corresponds to diatropic/paratropic ring currents. In the DIAL representation, diatropic regions are shown in blue and paratropic in red. Bond current strengths are given as percentages relative to a reference value of 12 nA T^{-1}.

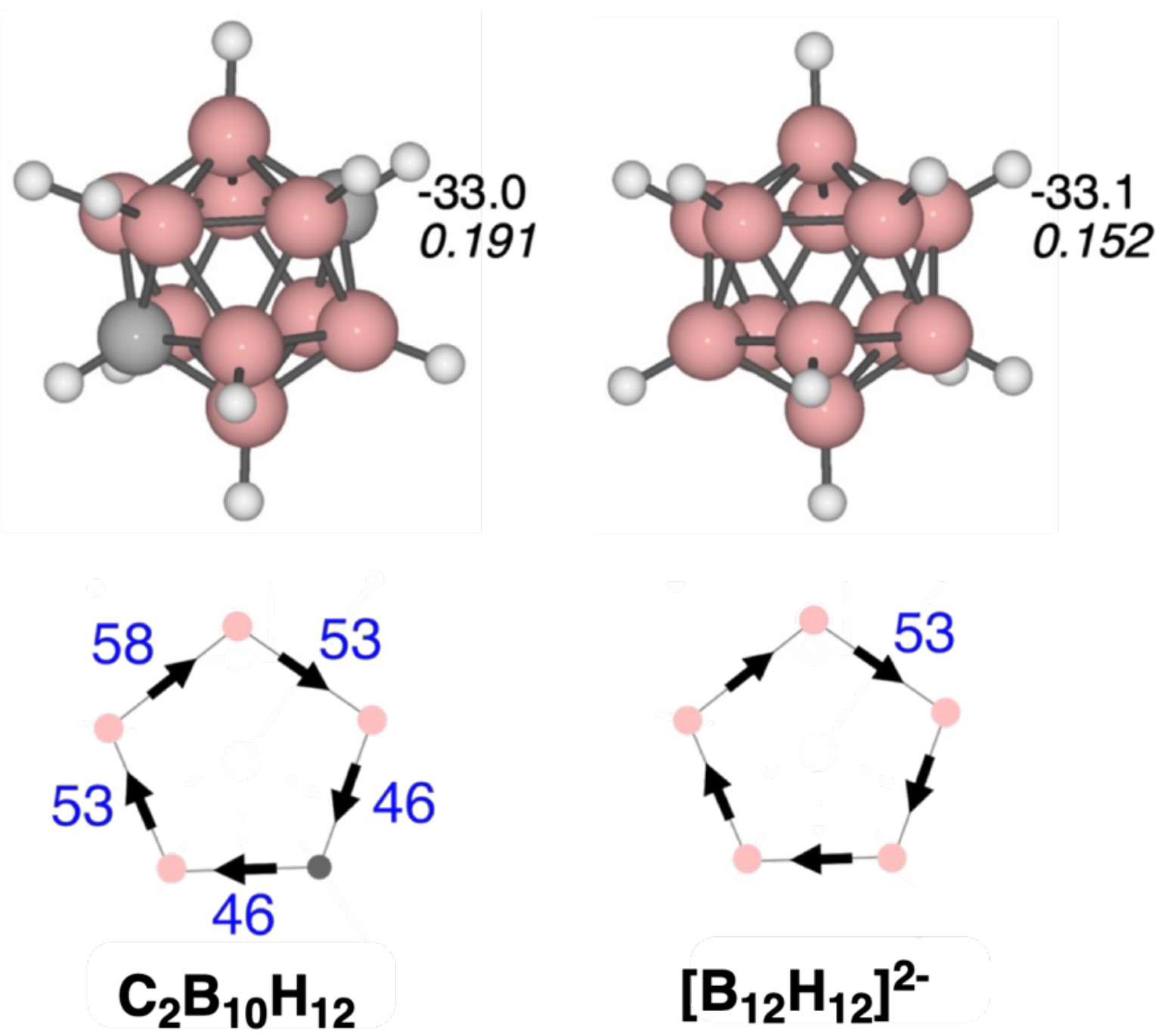

Figure S8. NICS (top value, in ppm), $MCI^{1/N}$ (bottom value, in italics, in a.u.), and bond current strengths (given as percentages relative to a reference value of 12 nA T^{-1}) of reference *closo* clusters $C_2B_{10}H_{12}$ and $[B_{12}H_{12}]^{2-}$.

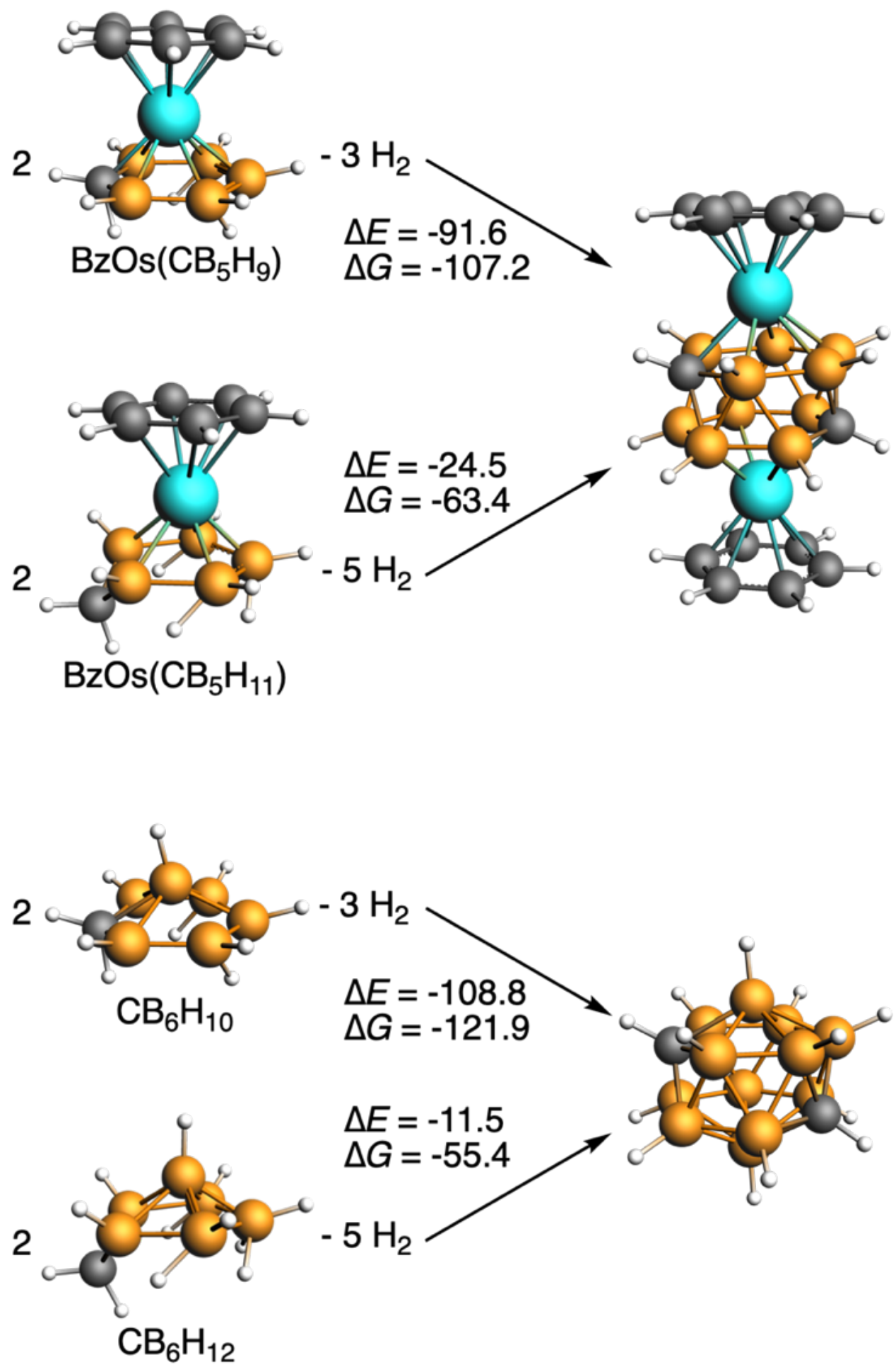

Figure S9. Formation energies (in kcal mol^{-1}) of $BzOs(C_2B_{10}H_{12})OsBz$ and $C_2B_{12}H_{14}$.

With the aim of further exploring the similarity between the CB_5 rings in the *nido* and *closo* structures, we examined the influence of the pre-organization of the CB_5 ring in the *nido* complex $BzOs(CB_5H_9)$, which is planar, compared with the non-planar CB_5 ensemble in its *arachno* precursor, $BzOs(CB_5H_{11})$, considering their transformation into $BzOs(C_2B_{10}H_{12})OsBz$. The pathway involving the pre-organized *nido* structure gives a more favorable ΔG value than that involving the *arachno* precursor (−107.2 vs −63.4 kcal mol^{-1}; Figure S9). However, these formation energies should not be regarded as a direct or quantitative measure of aromaticity, since the compared processes involve species with different hydrogenation states and, consequently, different numbers of H_2 molecules. Rather, the energetic trend can be viewed as qualitative support for the greater structural and electronic compatibility of the pre-organized *nido* CB_5 framework with the corresponding unit in the final *closo* structure. This observation is consistent with, but does not independently demonstrate, the 3D aromatic character assigned to both structures on the basis of the aromaticity descriptors discussed above.

The question that remains is: since the $BzOs^{2+}$ fragment contributes two electrons according to Polyhedral Skeletal Electron Pair Theory (PSEPT), what would happen if it were replaced by a simpler fragment that also contributes 2e−? Experimentally, the generation of carboranes with 13 and 14 vertices posed a significant challenge that was overcome by reducing the *closo* precursor to the *nido* dianion $[C_2B_{10}H_{12}]^{2-}$, followed by capitation through treatment with a BRX_2 compound. However, this was not sufficient, and it was necessary to

tether the cage carbon atoms to retain their adjacency during reduction. This approach led to the first example of a *closo* derivative of $C_2B_{11}H_{13}$,[1] and the first example derived from $C_2B_{12}H_{14}$.[2] It is worth noting that neither $C_2B_{11}H_{13}$ nor $C_2B_{12}H_{14}$ has been obtained in its naked form, although computationally both are feasible.

As observed for the metallacarboranes, the pathway involving the pre-organized structure yields a more negative ΔG value (−121.9 kcal mol^{-1}) than that involving the non-pre-organized structure (−55.4 kcal mol^{-1}; Figure S9). Again, because these transformations differ in hydrogenation state and H_2 stoichiometry, the magnitude of this energetic difference cannot be interpreted as an unambiguous measure of aromatic stabilization or used to establish the dimensionality of aromaticity. Nevertheless, the trend is compatible with the proposed geometric and electronic relationship between the six-membered units in *nido* CB_6H_{10} and *closo* $C_2B_{12}H_{14}$. Their assignment to the same type of 3D aromaticity therefore relies primarily on the electronic and magnetic aromaticity descriptors discussed above, with the formation-energy comparison providing only complementary qualitative information. For completeness, the fully boron central clusters, i.e., $B_{12}H_{12}$ instead of $C_2B_{10}H_{12}$, display an analogous qualitative energetic trend (Figure S10), subject to the same limitations in its interpretation.

[1] A. Burke, D. Ellis, B. T. Giles, B. E. Hodson, S. A. Macgregor, G. M. Rosair, A. J. Welch, *Angew. Chem. Int. Ed.* **2003**, *42*, 225-228.
[2] L. Deng, H.-S. Chan, Z. Xie, *Angew. Chem. Int. Ed.* **2005**, *44*, 2128-2131.

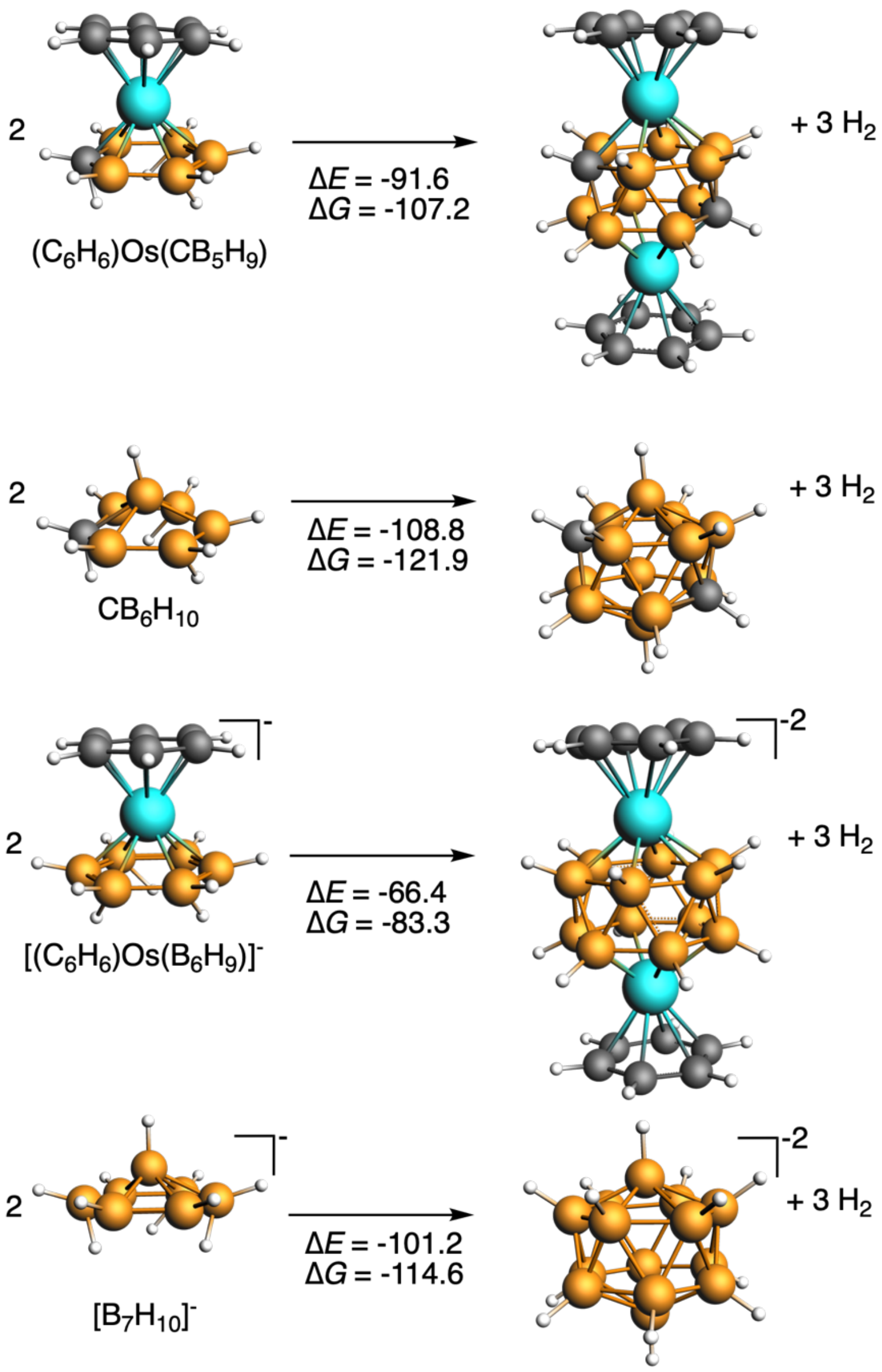

Figure S10. Formation energies (in kcal mol^{-1}) of $[BzOs(B_{12}H_{12})OsBz]^{2-}$ and $[B_{14}H_{14}]^{2-}$.

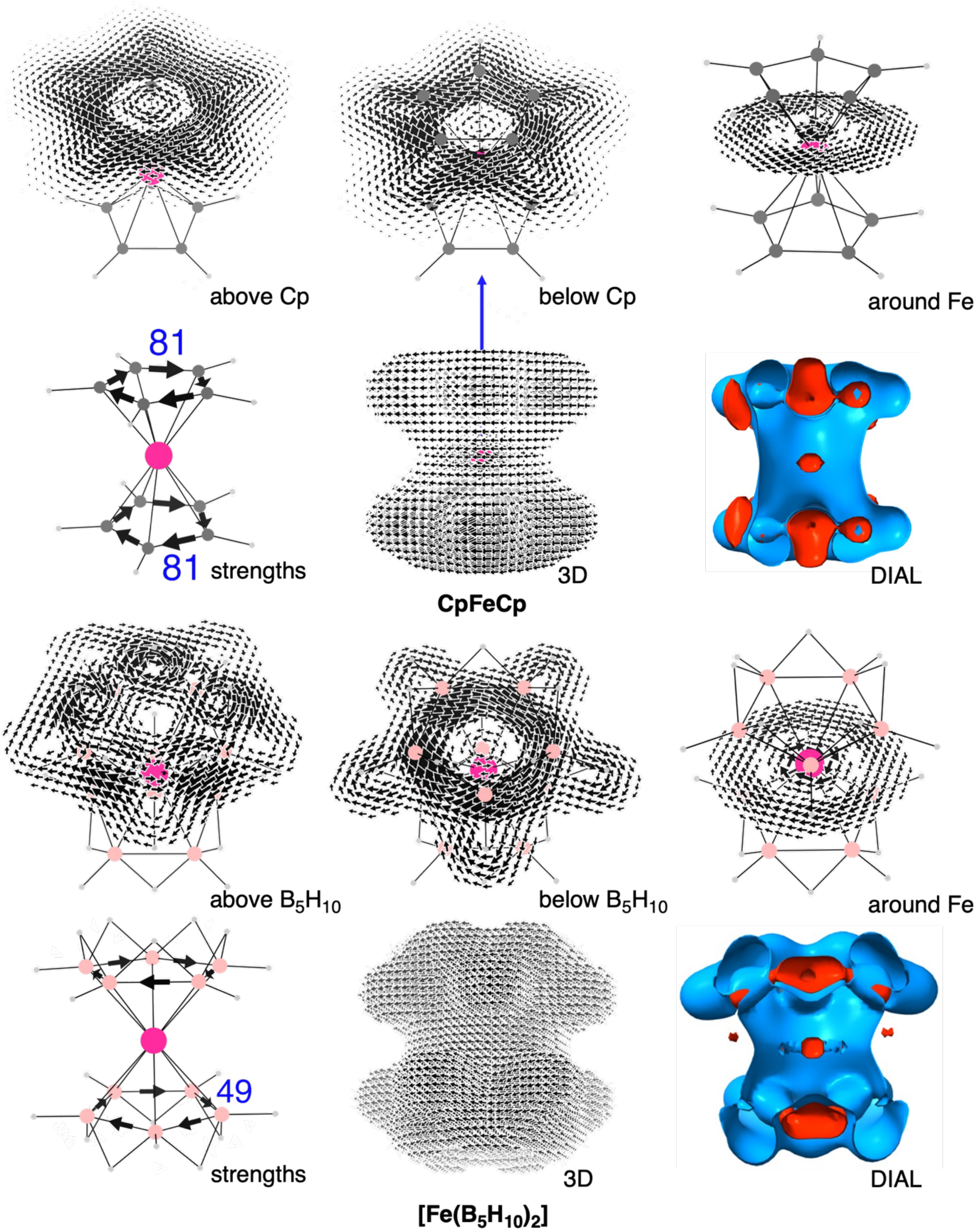

Figure S11. Current density maps of CpFeCp and $[Fe(B_5H_{10})_2]$. Clockwise/anticlockwise circulation corresponds to diatropic/paratropic ring currents. In the DIAL representation, diatropic regions are shown in blue and paratropic in red. Bond current strengths are given as percentages relative to a reference value of 12 nA T^{-1}.

Table S2. Cartesian coordinates (in Å) and ADF electronic energies (in kcal mol^{-1}) of all systems under analysis. Computed at ZORA-BLYP-D3(BJ)/TZ2P in toluene (COSMO).

C6H6CrC6H6 (-3537.9)

1	Cr	-0.01154309	-0.43592818	0.01997576
2	C	1.26875165	-0.95226805	-1.64990433
3	C	1.20070677	-1.29288403	1.59865245
4	H	0.07051940	-2.76708376	-1.81275335
5	C	-1.22386191	0.42080210	-1.55819276
6	C	1.17274516	0.12259083	1.74707227
7	H	-2.24218063	0.60932066	1.67473321
8	H	-2.12703553	-1.55655993	-1.73204793
9	C	1.24094891	0.46338044	-1.50233116
10	H	-2.19267435	-1.88689789	1.41276520
11	H	0.00436715	-3.09673151	1.33125305
12	H	2.21893742	-1.48139327	-1.63366245
13	H	2.15267290	-1.81187451	1.51232508
14	C	-0.00538241	1.14988316	-1.45634279
15	H	-2.17590911	0.93967060	-1.47195486
16	H	2.10365537	0.68452894	1.77399128
17	H	-0.09367934	1.89472893	1.85488586
18	H	2.16961890	1.01478137	-1.37352889
19	H	-0.02761747	2.22490891	-1.29249627
20	C	0.05030106	-1.68130336	-1.75135068
21	C	-1.19597050	-0.99483740	-1.70563615
22	C	-0.07357499	0.80902440	1.79292101
23	C	-1.29204461	0.08011602	1.69084723
24	C	-1.26417386	-1.33535839	1.54216520
25	C	-0.01778121	-2.02179017	1.49586644

CpOsCp (-2962.4)

1	Os	-0.00138180	-0.40805964	-0.00002060
2	C	1.23152376	-0.41049647	1.85097717
3	C	1.22150114	-0.41379133	-1.85747451
4	C	0.38407427	-1.57700458	1.85165930
5	C	0.38405947	0.75596629	1.85618439
6	C	0.37405069	0.75268049	-1.86022909
7	C	0.37403593	-1.58027250	-1.85170193
8	C	-0.98719273	-1.13145033	1.85755031
9	C	-0.98718748	0.31039664	1.86038793
10	C	-0.99721240	0.30715758	-1.85639483
11	C	-0.99724177	-1.13469329	-1.85106990
12	H	2.31371097	-0.41050522	1.86442165
13	H	2.30360616	-0.41385500	-1.87687672
14	H	0.71860376	-2.60623261	1.86504057
15	H	0.71855650	1.78511539	1.87477971
16	H	0.70843826	1.78179605	-1.88251486
17	H	0.70842621	-2.60954646	-1.86506685
18	H	-1.86260966	-1.76755958	1.87719046
19	H	-1.86258709	0.94641282	1.88331601
20	H	-1.87269642	0.94315967	-1.87551366
21	H	-1.87274541	-1.77081666	-1.86464466

Cp*OsB6H11 (-5185.5)

1	C	-1.45776448	-0.37709296	-1.15930520
2	C	-1.45730539	0.99955429	-0.72084480
3	C	-1.45757755	1.00356002	0.72210416
4	C	-1.46366688	-0.36666334	1.17389142
5	C	-1.46711388	-1.22147984	0.01274773
6	B	1.79964326	0.24636752	-1.71946303

7	B	1.78841111	-1.40760324	-1.03037798
8	B	1.81712178	-1.56477469	0.76492247
9	B	1.87101606	-0.14075169	1.79444118
10	B	1.88072272	1.35982050	1.16877246
11	B	1.83712726	1.63104524	-0.56726577
12	Os	0.49280765	-0.00573567	-0.01451142
13	H	1.46686562	0.41417109	-2.85180109
14	H	1.44623780	-2.32592588	-1.70939805
15	H	1.51471214	2.36256798	1.71100152
16	H	1.49562007	-0.45764850	2.88642974
17	H	2.85240016	1.69030002	0.32493089
18	H	2.70337235	-0.74298493	-1.74721288
19	H	2.72336331	-1.85531494	-0.22275751
20	H	2.83496548	-0.98520166	1.44026217
21	H	2.74386073	1.12253759	-1.46354427
22	H	1.53442082	-2.63744204	1.20092116
23	H	1.57204374	2.69908176	-1.02450402
24	C	-1.56033790	-0.82148343	2.60042997
25	H	-1.07922137	-0.11122303	3.27793647
26	H	-2.61406152	-0.91652301	2.89819194
27	H	-1.07987399	-1.79349118	2.74152528
28	C	-1.55216074	2.21732785	1.59927935
29	H	-2.60549430	2.46273265	1.79536775
30	H	-1.05795258	2.05329475	2.56061003
31	H	-1.08380155	3.08584405	1.12889438
32	C	-1.56413564	2.20695717	-1.60650651
33	H	-2.61898994	2.45534583	-1.78981622
34	H	-1.08741446	3.07840350	-1.14948134
35	H	-1.08635399	2.03349117	-2.57495080
36	C	-1.56643167	-0.84811905	-2.58088732
37	H	-1.08029823	-1.81841758	-2.71671150
38	H	-2.62168974	-0.95605239	-2.86775801
39	H	-1.10077982	-0.13972586	-3.27154903
40	C	-1.58078337	-2.71830249	0.01924626
41	H	-1.11154116	-3.14849840	0.90832961
42	H	-2.63704024	-3.02198530	0.01359106
43	H	-1.10038166	-3.15747490	-0.85970523

CpOsC6H6 (-3145.5)

1	Os	0.10682317	-0.46825609	-0.08995336
2	C	1.27537058	-0.40651406	1.81556970
3	H	2.35664633	-0.38754957	1.84792501
4	C	0.45031500	-1.58467860	1.81482838
5	C	0.41077989	0.74142264	1.76983919
6	H	-1.83444852	0.89392574	1.70866626
7	H	0.80185793	-2.60739529	1.84713765
8	C	-0.92416131	-1.16441314	1.76847720
9	C	-0.94875094	0.27347163	1.74066408
10	H	0.72715010	1.77606617	1.76280315
11	H	-1.78830014	-1.81539868	1.76004275
12	C	0.20889913	0.90588330	-1.87914001
13	C	-1.05028015	0.23552889	-1.89311217
14	C	-1.10036687	-1.18960994	-1.85204251
15	C	0.10857155	-1.94401462	-1.79481137
16	C	1.36715012	-1.27319651	-1.77912547
17	C	1.41740865	0.15116718	-1.82186765
18	H	-1.96980080	0.81013658	-1.88626749
19	H	-2.05816883	-1.69615816	-1.80938571
20	H	0.06908720	-3.02385726	-1.70402901
21	H	2.28308962	-1.84424884	-1.67467723

22	H	2.37130817	0.66202204	-1.75155440
23	H	0.24439621	1.98954324	-1.85558144

C6H6 (-1681.4)

1	C	0.00000000	1.40057834	0.00000000
2	C	1.21293642	0.70028917	0.00000000
3	C	1.21293642	-0.70028917	0.00000000
4	C	-0.00000000	-1.40057834	0.00000000
5	C	-1.21293642	-0.70028917	0.00000000
6	C	-1.21293642	0.70028917	0.00000000
7	H	2.15497934	1.24417791	0.00000000
8	H	2.15497934	-1.24417791	0.00000000
9	H	-0.00000000	-2.48835581	0.00000000
10	H	-2.15497934	-1.24417791	0.00000000
11	H	-2.15497934	1.24417791	0.00000000
12	H	0.00000000	2.48835581	0.00000000

C5H5- (-1423.1)

1	C	0.97919271	0.71142514	0.00000000
2	C	0.97919271	-0.71142514	0.00000000
3	C	-0.37401833	-1.15111006	0.00000000
4	C	-1.21034875	0.00000000	0.00000000
5	C	-0.37401833	1.15111006	0.00000000
6	H	1.85959617	-1.35107570	0.00000000
7	H	-2.29858728	0.00000000	0.00000000
8	H	1.85959617	1.35107570	0.00000000
9	H	-0.71030253	-2.18608641	0.00000000
10	H	-0.71030253	2.18608641	0.00000000

Cp*OsCp* (-6662.2)

1	Os	0.00147923	-0.41055823	-0.00069930
2	C	1.22630925	-0.42051780	1.84857635
3	C	1.24028907	-0.40178941	-1.84142812
4	C	0.37918948	-1.59689477	1.83646668
5	C	0.36946911	0.74868449	1.85608196
6	C	0.39131802	0.77327101	-1.83491170
7	C	0.38502286	-1.57217293	-1.85037341
8	C	-1.00125909	-1.15461219	1.83295359
9	C	-1.00726053	0.29498162	1.84498107
10	C	-0.98872884	0.32899310	-1.84185112
11	C	-0.99258652	-1.12043689	-1.85128455
12	H	3.15575032	-1.29243421	1.44314998
13	H	3.08612233	-0.40414512	-2.96303420
14	H	1.82425348	-3.14294328	1.42735727
15	H	1.80274011	2.31306298	1.47672147
16	H	0.96136208	2.53288865	-2.95032009
17	H	0.94153324	-3.32193331	-2.98768861
18	H	-2.48725371	-2.26047814	2.94374951
19	H	-2.49915799	1.37364562	2.97450168
20	H	-2.02089344	2.17467577	-1.42525427
21	H	-2.03487988	-2.96523949	-1.45635299
22	C	-2.20773815	-1.99857017	-1.94068138
23	H	-3.06987699	-1.53374898	-1.45125127
24	H	-2.48273715	-2.19371940	-2.98832454
25	C	-2.19930112	1.21446628	-1.92028516
26	H	-2.47415461	1.42287787	-2.96541746
27	H	-3.06350151	0.74877713	-1.43533719
28	C	-2.22355895	1.17195429	1.92823122
29	H	-2.05193567	2.13580447	1.43774703
30	H	-3.08493044	0.70294065	1.44155040

31	C	-2.21044107	-2.04261400	1.90106171
32	H	-3.07435168	-1.57443351	1.41808556
33	H	-2.02939996	-2.99841139	1.39835540
34	C	0.84386352	-2.99937351	-1.93997116
35	H	1.81787921	-3.13387590	-1.45823147
36	H	0.13647982	-3.67519564	-1.44820848
37	C	0.85802119	2.19897609	-1.90669399
38	H	0.15232473	2.87286260	-1.40990433
39	H	1.83088060	2.32293326	-1.41978117
40	C	0.82556287	2.17645920	1.95131562
41	H	0.12052258	2.85207536	1.45589759
42	H	0.91525470	2.49740362	3.00028143
43	C	0.84775988	-3.02203412	1.90758797
44	H	0.94421126	-3.35848883	2.95107440
45	H	0.14663435	-3.69587660	1.40427824
46	C	2.74021944	-0.40552229	-1.91822905
47	H	3.16476146	0.47550867	-1.42590554
48	H	3.16034537	-1.29090482	-1.42982623
49	C	2.72562237	-0.41542386	1.93793802
50	H	3.14858145	0.47366177	1.45885980
51	H	3.06289491	-0.42354439	2.98548575

CpFeCp (-2961.5)

1	Fe	-0.00042668	-0.41012247	-0.00001126
2	C	1.22616772	-0.41079518	1.66118785
3	C	1.21806881	-0.41340450	-1.66774327
4	C	0.38171196	-1.57308100	1.66258269
5	C	0.38174366	0.75148740	1.66525132
6	C	0.37360023	0.74885628	-1.66932797
7	C	0.37363797	-1.57570553	-1.66259998
8	C	-0.98466161	-1.12912438	1.66744596
9	C	-0.98462762	0.30756527	1.66913394
10	C	-0.99276008	0.30490739	-1.66508133
11	C	-0.99274985	-1.13177693	-1.66090123
12	H	2.30891401	-0.41074565	1.63746913
13	H	2.30094417	-0.41330459	-1.65062355
14	H	0.71624844	-2.60289814	1.63975433
15	H	0.71630238	1.78137367	1.64646894
16	H	0.70821763	1.77878775	-1.65464130
17	H	0.70831929	-2.60547570	-1.63974634
18	H	-1.86076900	-1.76556300	1.65002113
19	H	-1.86068546	0.94412669	1.65430041
20	H	-1.86873517	0.94147946	-1.64610528
21	H	-1.86872842	-1.76818560	-1.63683430

CpRuCp (-2950.8)

1	Ru	-0.00155343	-0.40760816	-0.00003424
2	C	1.22827600	-0.41056112	1.85036296
3	C	1.21836054	-0.41385450	-1.85699452
4	C	0.38306883	-1.57402383	1.85053019
5	C	0.38302096	0.75281816	1.85656711
6	C	0.37310813	0.74956662	-1.86069448
7	C	0.37308086	-1.57727624	-1.85057855
8	C	-0.98465121	-1.12967672	1.85704544
9	C	-0.98464431	0.30843382	1.86080310
10	C	-0.99457680	0.30522983	-1.85675440
11	C	-0.99462046	-1.13285752	-1.85047114
12	H	2.31078831	-0.41040774	1.86084126
13	H	2.30080089	-0.41371891	-1.87350826
14	H	0.71766404	-2.60355263	1.86065552

15	H	0.71747977	1.78230376	1.87320399
16	H	0.70763272	1.77896477	-1.88104862
17	H	0.70771146	-2.60679449	-1.86063707
18	H	-1.86039757	-1.76585006	1.87386844
19	H	-1.86023699	0.94469844	1.88169174
20	H	-1.87014831	0.94162898	-1.87374964
21	H	-1.87043105	-1.76906123	-1.86109893

Welch[Os]2 (-6599.2)

1	Os	9.16197833	11.29224886	1.70449139
2	C	10.74547076	9.91469283	0.78421034
3	C	10.46290696	10.95541093	-0.14941276
4	B	7.25776044	11.72210539	0.49608698
5	C	7.31585366	10.10225735	1.02692155
6	B	7.88846575	9.57674297	2.54277679
7	B	8.27153614	10.82685805	3.77072588
8	C	11.23707573	11.60434594	2.51683927
9	B	7.66202665	12.98731767	1.67053542
10	C	10.95738414	12.64484217	1.58082887
11	B	8.16546406	12.53349949	3.32770810
12	H	10.59771025	8.87733030	0.50121206
13	C	11.13339895	10.23726903	2.11766510
14	H	10.10095226	10.70833559	-1.14248918
15	C	10.57681715	12.32126651	0.24279138
16	H	7.40633786	11.91085484	-0.67202984
17	B	5.96548670	12.53379219	1.38426115
18	B	5.78674120	10.82661512	0.94165423
19	H	7.52647489	9.40553609	0.22138573
20	B	6.15960910	9.55412827	2.14466125
21	H	8.42885025	8.51277424	2.56157656
22	B	6.68784356	10.01513821	3.76693791
23	H	9.03885377	10.52628624	4.63751731
24	B	6.82235618	11.75349689	4.18506287
25	H	11.45943056	11.85175059	3.54945303
26	H	7.96853426	14.07962333	1.30439797
27	C	6.55046726	12.89064965	2.94672225
28	H	10.96948598	13.67997880	1.90554743
29	H	8.76827607	13.37211996	3.92509187
30	H	11.27517318	9.44839262	2.84827497
31	H	10.29945838	13.11026591	-0.44776277
32	H	5.29626829	13.36869405	0.85749901
33	Os	4.92473062	11.26554914	2.98867040
34	H	5.08605113	10.50701516	0.03133930
35	H	5.67377219	8.48681121	1.92473084
36	H	6.46117075	9.25045162	4.65783376
37	H	6.63106147	12.13269044	5.30031485
38	H	6.20506064	13.85178109	3.31486986
39	H	3.06942075	13.68234116	2.80516369
40	H	2.81928051	9.45167431	1.85191305
41	H	3.79294105	13.11278530	5.14078554
42	H	2.56797654	11.85479022	1.16299171
43	H	4.03180168	10.71259855	5.83036709
44	C	3.51484127	12.32234867	4.45138834
45	C	3.37343100	9.91754793	3.90817328
46	C	2.81673203	11.60798623	2.18970845
47	C	3.64827483	10.95872570	4.84595319
48	C	3.10401505	12.64540950	3.12424342
49	H	3.54599698	8.88229562	4.18290554
50	C	2.96043958	10.24210777	2.58117558

Welch[Ru]2 (-6559.6)

1	Ru	9.15568339	11.28875354	1.70219879
2	C	10.75386935	9.91692368	0.78809001
3	C	10.47545322	10.95410109	-0.14519939
4	B	7.25737161	11.71621446	0.50360319
5	C	7.30590159	10.10071707	1.02633833
6	B	7.87965136	9.58389092	2.53417565
7	B	8.26682559	10.83232215	3.76380587
8	C	11.23166141	11.60199733	2.51749272
9	B	7.66397660	12.98574932	1.66929906
10	C	10.95467379	12.63906983	1.58198635
11	B	8.16832989	12.53538619	3.32215615
12	H	10.61408622	8.87942647	0.50305095
13	C	11.13474910	10.23889758	2.11912612
14	H	10.12458765	10.70577469	-1.14148950
15	C	10.58434936	12.31601376	0.24596130
16	H	7.41793869	11.90547974	-0.66280505
17	B	5.97110427	12.53245915	1.38970496
18	B	5.78437222	10.82944672	0.94250837
19	H	7.52062657	9.40256225	0.22262934
20	B	6.15421367	9.55924838	2.14245884
21	H	8.43245773	8.52678317	2.55325309
22	B	6.68755053	10.02138002	3.75745855
23	H	9.04126791	10.52890648	4.62347337
24	B	6.82395931	11.75993624	4.17239884
25	H	11.45349648	11.85022016	3.54948736
26	H	7.98497118	14.07250013	1.29618503
27	C	6.55893710	12.89542537	2.94300003
28	H	10.96800979	13.67420850	1.90511571
29	H	8.78615240	13.36848668	3.91168133
30	H	11.28108578	9.44992987	2.84875876
31	H	10.31585559	13.10470947	-0.44850743
32	H	5.29373954	13.36372312	0.86775858
33	Ru	4.93018154	11.27319958	2.98696242
34	H	5.07145610	10.51390755	0.04007845
35	H	5.65364558	8.49701457	1.93382949
36	H	6.45075989	9.26317339	4.65252588
37	H	6.61839984	12.13158106	5.28749626
38	H	6.21071393	13.85580213	3.31156439
39	H	3.04321148	13.67633527	2.82074991
40	H	2.82598618	9.45448273	1.85328896
41	H	3.77461147	13.10457211	5.14832829
42	H	2.55252814	11.85324467	1.17484472
43	H	4.02845833	10.70822328	5.82986383
44	C	3.50586633	12.31406938	4.45515396
45	C	3.38231798	9.91807065	3.90609293
46	C	2.81195524	11.60544194	2.19818270
47	C	3.64913049	10.95457136	4.84436198
48	C	3.09152129	12.63844887	3.13344831
49	H	3.55746553	8.88297387	4.17778703
50	C	2.96632856	10.24377377	2.58395523

Welch[Fe]2 (-6559.1)

1	Fe	9.06262805	11.29179982	1.73351538
2	C	10.48128331	9.92217298	0.87942216
3	C	10.19887073	10.94649660	-0.05701121
4	B	7.27076038	11.71189804	0.52908482
5	C	7.32669465	10.11523102	1.04170592
6	B	7.88679249	9.60434058	2.53201231
7	B	8.26168820	10.83761207	3.74998500

8	C	10.98120157	11.61436682	2.58102399
9	B	7.66382905	12.96766118	1.68321859
10	C	10.70311117	12.63906661	1.64172681
11	B	8.16079144	12.52333208	3.31273716
12	H	10.29794550	8.88538602	0.61867332
13	C	10.87063914	10.25392103	2.19985576
14	H	9.80175432	10.69200936	-1.03382837
15	C	10.31292965	12.30659081	0.32078853
16	H	7.43238709	11.90791884	-0.63587388
17	B	5.97202453	12.51271956	1.41018199
18	B	5.79540372	10.82919275	0.96395438
19	H	7.54340929	9.41839475	0.23666728
20	B	6.16017709	9.57625929	2.14543981
21	H	8.44197734	8.54866837	2.55697382
22	B	6.68386677	10.03092723	3.74588625
23	H	9.03792436	10.53705834	4.60854188
24	B	6.81155921	11.75085188	4.15764413
25	H	11.17899948	11.87069214	3.61541828
26	H	7.98647886	14.05315850	1.30969159
27	C	6.54398519	12.87195612	2.94615786
28	H	10.68946144	13.67406173	1.96382936
29	H	8.78038790	13.35470026	3.90251841
30	H	10.98533775	9.47348436	2.94322483
31	H	10.00498312	13.08749824	-0.36529580
32	H	5.29243872	13.33872778	0.88363244
33	Fe	5.02351172	11.26958277	2.95844106
34	H	5.08261761	10.51350780	0.06188569
35	H	5.65774680	8.51450082	1.93729290
36	H	6.44523953	9.27344348	4.64025843
37	H	6.60572834	12.11747574	5.27412992
38	H	6.19344574	13.83227406	3.31428022
39	H	3.36868056	13.67386733	2.73671149
40	H	3.09762839	9.47432345	1.75690189
41	H	4.08922030	13.08538965	5.05467785
42	H	2.86527238	11.86936296	1.08806214
43	H	4.31989645	10.69267038	5.72100657
44	C	3.77604970	12.30367166	4.37088752
45	C	3.62666171	9.92117397	3.81341448
46	C	3.08324594	11.61455962	2.11892655
47	C	3.90848988	10.94600135	4.75083074
48	C	3.36864781	12.63723999	3.05617262
49	H	3.82404695	8.88652441	4.06901813
50	C	3.21359549	10.25580512	2.49926903

CpOsB5H10 (-3114.8)

1	Os	-0.01443570	-0.40433939	-0.28817465
2	C	1.22194929	-0.41004527	1.68230028
3	B	1.51697996	-0.40244356	-1.76698313
4	C	0.37662556	-1.56821607	1.68827199
5	C	0.38167026	0.75185820	1.69095397
6	B	0.45018218	1.06797660	-1.75717579
7	B	0.44839877	-1.87146222	-1.76418981
8	C	-0.98613927	-1.12206992	1.70113248
9	C	-0.98284463	0.31173678	1.70278880
10	B	-1.27714717	0.50723980	-1.74274690
11	B	-1.27834882	-1.30958774	-1.74354817
12	H	2.30396234	-0.41254819	1.67958997
13	H	2.70055966	-0.40447788	-1.62708989
14	H	0.70849654	-2.59807759	1.69076410
15	H	0.71815689	1.78020737	1.69596034

16	H	0.81554297	2.19256994	-1.60573291
17	H	0.81515534	-2.99586653	-1.61833512
18	H	-1.86291253	-1.75596154	1.71367051
19	H	-1.85667233	0.94969648	1.71742234
20	H	-2.23030443	1.20358694	-1.57829461
21	H	-2.23225502	-2.00717747	-1.58681152
22	H	-0.53905706	-1.94159092	-2.67505381
23	H	1.27558163	-1.35404388	-2.69251415
24	H	1.27900150	0.55587487	-2.68550229
25	H	-0.53523516	1.14239653	-2.67030449
26	H	-1.66202247	-0.39935987	-2.66023466

CpOsB6H11 (-3332.1)

1	C	-1.48966049	-0.37573165	-1.15226879
2	C	-1.48544142	0.99306613	-0.71838284
3	C	-1.47898090	0.99836477	0.71530667
4	C	-1.48788846	-0.36238715	1.16585660
5	C	-1.50020274	-1.21365280	0.01340038
6	B	1.78841758	0.24449466	-1.72052735
7	B	1.77985146	-1.40808107	-1.03193435
8	B	1.80631456	-1.56587646	0.76227250
9	B	1.85048765	-0.14153455	1.79586321
10	B	1.85756321	1.35884560	1.17072480
11	B	1.82191921	1.62913865	-0.56858712
12	Os	0.47554261	-0.00959690	-0.01947657
13	H	1.45501751	0.41300534	-2.85182150
14	H	1.43953167	-2.32627247	-1.71046556
15	H	1.48427521	2.36043872	1.70953965
16	H	1.46926217	-0.46147512	2.88450620
17	H	2.82909382	1.68725864	0.32908578
18	H	2.69239235	-0.74048776	-1.74631032
19	H	2.71231287	-1.85206953	-0.21963878
20	H	2.81564985	-0.98026947	1.44018690
21	H	2.72755923	1.12536256	-1.45900643
22	H	1.52248222	-2.63726164	1.19854401
23	H	1.55246065	2.69682090	-1.02263061
24	H	-1.47665492	-0.68840819	2.19737251
25	H	-1.51511585	-2.29539956	0.02115931
26	H	-1.50093792	-0.71628138	-2.17928452
27	H	-1.49024926	1.86530282	-1.35832094
28	H	-1.46205712	1.87551273	1.34823924

[B12H12]2- (-2864.8)

1	B	1.44478532	0.46943921	0.75956859
2	H	2.46669197	0.80147680	1.29681672
3	B	0.89292643	-1.22900780	0.75956859
4	H	1.52449948	-2.09829352	1.29681672
5	B	1.44478532	-0.46943921	-0.75956859
6	H	2.46669197	-0.80147680	-1.29681672
7	B	-0.00000000	-0.00000000	-1.69844701
8	H	0.00000000	0.00000000	-2.89977033
9	B	0.00000000	0.00000000	1.69844701
10	H	-0.00000000	-0.00000000	2.89977033
11	B	-1.44478532	0.46943921	0.75956859
12	H	-2.46669197	0.80147680	1.29681672
13	B	-0.89292643	-1.22900780	0.75956859
14	H	-1.52449948	-2.09829352	1.29681672
15	B	0.00000000	-1.51913718	-0.75956859
16	H	-0.00000000	-2.59363343	-1.29681672
17	B	-0.00000000	1.51913718	0.75956859

18	H	0.00000000	2.59363343	1.29681672
19	B	0.89292643	1.22900780	-0.75956859
20	H	1.52449948	2.09829352	-1.29681672
21	B	-1.44478532	-0.46943921	-0.75956859
22	H	-2.46669197	-0.80147680	-1.29681672
23	B	-0.89292643	1.22900780	-0.75956859
24	H	-1.52449948	2.09829352	-1.29681672

C2B10H12 (-2843.0)

1	B	1.43720639	0.47307219	0.76544689
2	H	2.49569426	0.74880794	1.21159672
3	B	0.88465953	-1.22749216	0.76544689
4	H	1.57892080	-2.07273065	1.21159672
5	C	1.30512626	-0.42406123	-0.68614548
6	H	2.22632773	-0.72337773	-1.17044976
7	B	-0.00938005	0.00304776	-1.69563296
8	H	0.09555018	-0.03104613	-2.87177230
9	B	0.00938005	-0.00304776	1.69563296
10	H	-0.09555018	0.03104613	2.87177230
11	C	-1.30512626	0.42406123	0.68614548
12	H	-2.22632773	0.72337773	1.17044976
13	B	-0.88465953	-1.23358769	0.75558412
14	H	-1.57892080	-2.01063837	1.31206413
15	B	-0.00938005	-1.51798323	-0.75558412
16	H	0.09555018	-2.55470697	-1.31206413
17	B	0.00938005	1.51798323	0.75558412
18	H	-0.09555018	2.55470697	1.31206413
19	B	0.88465953	1.23358769	-0.75558412
20	H	1.57892080	2.01063837	-1.31206413
21	B	-1.43720639	-0.47307219	-0.76544689
22	H	-2.49569426	-0.74880794	-1.21159672
23	B	-0.88465953	1.22749216	-0.76544689
24	H	-1.57892080	2.07273065	-1.21159672

Os(B5H10)2 (-3238.5)

1	Os	-0.01957034	-0.38662441	-0.01335923
2	B	1.53482752	-0.38004312	1.54824332
3	B	1.51693101	-0.39116112	-1.59288067
4	B	0.46332299	-1.84745455	1.56462533
5	B	0.47040325	1.09168822	1.54549493
6	B	0.44547816	1.07528664	-1.59467614
7	B	0.45357549	-1.86376684	-1.57868635
8	B	-1.26379214	-1.28075993	1.57148572
9	B	-1.25924741	0.53644500	1.55616030
10	B	-1.28052395	0.50919224	-1.58308471
11	B	-1.27580373	-1.30763359	-1.57060008
12	H	2.71625760	-0.38187906	1.42349132
13	H	2.69944784	-0.38786383	-1.48057945
14	H	0.82295586	-2.97391482	1.45034287
15	H	0.83981204	2.21357318	1.41826040
16	H	0.80748680	2.20116271	-1.48406609
17	H	0.82383289	-2.98564399	-1.45526596
18	H	-2.22176253	-1.97446683	1.46079200
19	H	-2.21398748	1.23338542	1.43861190
20	H	-2.23752645	1.20226592	-1.46210881
21	H	-2.22967913	-2.00378249	-1.44266547
22	H	-0.52763434	-1.93701049	-2.48236336
23	H	1.27312941	-1.34393037	-2.49737501
24	H	1.26962995	0.55154893	-2.50650753
25	H	-0.53526569	1.13076155	-2.50091434

26	H	-1.64570931	-0.40553701	-2.48541634
27	H	1.29824939	0.57051353	2.45709476
28	H	1.29941117	-1.32442761	2.46514213
29	H	-0.50778449	-1.90191749	2.48076989
30	H	-1.61998916	-0.36580212	2.47547314
31	H	-0.50004273	1.16273123	2.46099283

Ru(B5H10)2 (-3204.8)

1	Ru	-0.01820243	-0.38642384	-0.01333209
2	B	1.52842733	-0.38101159	1.54146873
3	B	1.51067681	-0.39039627	-1.58576590
4	B	0.46057966	-1.84125566	1.55797815
5	B	0.46953335	1.08556315	1.53722755
6	B	0.44260371	1.06925986	-1.58741024
7	B	0.45276800	-1.85724710	-1.57088252
8	B	-1.25915017	-1.27603652	1.56264320
9	B	-1.25287428	0.53309808	1.54870631
10	B	-1.27566504	0.50433713	-1.57432238
11	B	-1.26930125	-1.30448368	-1.56298243
12	H	2.70857076	-0.38627009	1.40915855
13	H	2.69213773	-0.38580310	-1.46608472
14	H	0.82036543	-2.96635493	1.43517742
15	H	0.83843759	2.20620109	1.40183223
16	H	0.80364961	2.19430352	-1.46872594
17	H	0.82276005	-2.97799040	-1.43968883
18	H	-2.21708723	-1.96773022	1.44320661
19	H	-2.20632834	1.22964020	1.42225366
20	H	-2.23243653	1.19568716	-1.44484821
21	H	-2.22167141	-2.00049047	-1.42605201
22	H	-0.52721361	-1.93864053	-2.47303096
23	H	1.27516098	-1.34463048	-2.48830445
24	H	1.26955614	0.55294378	-2.49823664
25	H	-0.53757189	1.13227503	-2.49107314
26	H	-1.64754895	-0.40671765	-2.47533712
27	H	1.30144040	0.57269186	2.44718850
28	H	1.29749864	-1.32473307	2.45839578
29	H	-0.51077172	-1.90447569	2.46996425
30	H	-1.62170263	-0.36485494	2.46584437
31	H	-0.50020820	1.16448031	2.45146354

Fe(B5H10)2 (-3206.5)

1	Fe	-0.02033154	-0.38651284	-0.01291297
2	B	1.50350550	-0.39189388	1.38826622
3	B	1.48775473	-0.37911908	-1.43126077
4	B	0.44195556	-1.82295261	1.40558243
5	B	0.47080043	1.05949098	1.38464039
6	B	0.42589912	1.05050473	-1.43495582
7	B	0.45654461	-1.83114162	-1.41695073
8	B	-1.24790321	-1.25414004	1.41202948
9	B	-1.22954283	0.52770387	1.39630279
10	B	-1.26216235	0.48229545	-1.42332482
11	B	-1.24333989	-1.29895074	-1.41130724
12	H	2.68091848	-0.40442693	1.23801086
13	H	2.66662972	-0.36578844	-1.29478861
14	H	0.79207067	-2.94852102	1.26595829
15	H	0.84856645	2.17443745	1.23196485
16	H	0.77863483	2.17581447	-1.30165695
17	H	0.83405209	-2.94645558	-1.26848784
18	H	-2.20882749	-1.93781603	1.27722515
19	H	-2.17610911	1.22970952	1.25424465

20	H	-2.22173975	1.16606566	-1.28129160
21	H	-2.18867034	-2.00013309	-1.25915605
22	H	-0.51208840	-1.93739524	-2.32653305
23	H	1.27986552	-1.33170653	-2.34001906
24	H	1.25992729	0.55919978	-2.35107478
25	H	-0.54510048	1.12300439	-2.34637461
26	H	-1.63996945	-0.41893603	-2.33101911
27	H	1.30193928	0.55866560	2.30063523
28	H	1.28694692	-1.33161637	2.31098266
29	H	-0.51929817	-1.89439360	2.32628513
30	H	-1.61637002	-0.35330576	2.32107105
31	H	-0.48812567	1.16324847	2.30434614
